# m^6^A‐Mediated TMCO3 Promotes Hepatocellular Carcinoma Progression by Facilitating the Membrane Translocation and Activation of AKT

**DOI:** 10.1002/advs.202504187

**Published:** 2025-04-26

**Authors:** Xinxin Li, Mengzhen Han, He Zhu, Yonglong Pan, Chen Su, Yachong Liu, Zhibin Liao, Bixiang Zhang, Xiaoping Chen

**Affiliations:** ^1^ Hepatic Surgery Center Tongji Hospital Tongji Medical College Huazhong University of Science and Technology Wuhan Hubei 430030 China; ^2^ Hubei Key Laboratory of Hepato‐Pancreato‐Biliary Diseases Wuhan Hubei 430030 China; ^3^ Department of General Surgery Ezhou Central Hospital Ezhou Hubei 436099 China; ^4^ Key Laboratory of Organ Transplantation Ministry of Education Wuhan Hubei 430030 China

**Keywords:** AKT, HCC, m^6^A, MK2206, TMCO3

## Abstract

The transmembrane and coiled‐coil domains 3 (TMCO3) are highly expressed in many tumors. However, the underlying mechanisms governing the way in which TMCO3 affects the progression of hepatocellular carcinoma (HCC) remain unclear. This study screens out the molecule TMCO3 with high N6‐methyladenosine (m^6^A) modification level in tumor samples compared to the adjacent non‐cancerous tissues of three pairs of HCC patients through Methylated RNA Immunoprecipitation Sequencing (MeRIP‐seq) and RNA sequencing (RNA‐seq). Subsequently, the oncogenic effect of TMCO3 in HCC is verified through in vivo and in vitro experiments. AlkB Homolog 5 (ALKBH5), an m^6^A demethylase of TMCO3 is then screened out. The following experiments demonstrate that TMCO3 can activate AKT directly through the Phosphatidylinositol‐3–Kinase (PI3K) pathway, thus promoting the progression of HCC. Meanwhile, the phosphorylation site on TMCO3: the 85^th^ amino acid‐serine, and mutation of this site can directly impair the activity and membrane translocation of AKT is found. Finally, the carcinogenic effect of TMCO3 is further elucidated in HCC through the orthotopic treatment model and the hydrodynamic tail vein injection treatment model. The findings can provide a potential target for targeted AKT treatment in patients with HCC and verify a possible prognostic marker in HCC.

## Graphical abstract

TMCO3 as a downstream target of ALKBH5 is found, which regulates its expression in an m^6^A‐dependent manner, and then a new phosphorylation site on TMCO3 is identified and determines the specific mechanism of TMCO3 with AKT. Moreover, the combined treatment of targeting the TMCO3 and inhibition of AKT in mice models with HCC achieves great results.

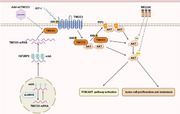

## 1 Introduction

HCC as the most common type of liver cancer is the fourth leading cause of cancer‐related death worldwide.^[^ ^1^ ^]^ The high mortality rate of HCC is mainly due to late‐stage HCC and a high rate of metastasis or recurrence. Despite the advanced surgical techniques currently available, there are still some patients with late‐stage HCC or metastasis that are difficult to cure clinically, necessitating the importance of investigating the molecular mechanisms of HCC progression.^[^ ^2^ ^]^

TMCO3 is a member of monovalent cation: proton antiporter 2 (CPA2) family of transporter proteins.^[^ ^3^ ^]^ Members of this family typically couple the export of monovalent cations, such as potassium or sodium, to the import of protons across cellular membranes.^[^ ^4^ ^]^ Members of this family have been poorly studied in cancer area, and tumor research on TMCO3 have been negligible. Hence, it is particularly important and urgent to explore the mechanism of TMCO3 in HCC.

RNA modifications have always occupied a significant position in the field of epigenetics, and the diversity and extensiveness of its modification types, especially research of the mechanism in tumors, are particularly important.^[^ ^5^ ^]^ m^6^A plays a crucial role in this process. As the most abundant type of mRNA modifications, m^6^A modifications regulated by “writers” (METTL3/METTL14/WTAP), “erasers” (FTO/ALKBH5), and “readers” (YTHDF1‐3/YTHDC1‐2) that respectively catalyze m^6^A methylation and demethylation, and recognize m^6^A‐modified RNA.^[^ ^6^ ^]^ To determine the regulator of TMCO3, our experiments confirmed that ALKBH5 is capable of demethylating the modification at 348^th^ site on TMCO3, and Insulin‐like Growth Factor 2 mRNA‐Binding Protein 2 (IGF2BP2） can recognize and stabilize TMCO3 mRNA.

Activation of the PI3K/AKT pathway is dependent on the stimulation from cytokines such as Insulin‐like Growth Factor 1 (IGF‐1), Epidermal Growth Factor (EGF), and others.^[^ ^7^ ^]^ Activation of AKT itself relies on the catalysis by molecules including the PI3K subunits, 3‐Phosphoinositide‐Dependent Protein Kinase‐1 (PDK1), and mammalian Target of Rapamycin Complex 2 (mTORC2).^[^ ^8^ ^]^ A prerequisite for AKT activation is its translocation from the cytoplasm to the membrane, where it aggregates. Following this aggregation, AKT binds to PtdIns(3,4,5)P_3_ (PIP_3_), starting the phosphorylation of AKT through the activation of the T308 site by PDK1, then mTORC2 completes AKT activation by stimulating the S473 site.^[^ ^9^ ^]^ Our research discovered that under the stimulation of IGF‐1, TMCO3 could promote the membrane accumulation of AKT and participate in its activation. Subsequently, we found that activation is due to the stimulation of the 85th serine residue on TMCO3, thus adding a new potential target for upstream regulatory molecules of AKT.

In summary, our research marked the first discovery that TMCO3 was highly expressed and exhibited a high m^6^A methylation level in HCC. The oncogenic effects of TMCO3 were ascertained through both in vivo and in vitro experiments, and ALKBH5 was also verified as a m^6^A demethylase to downregulate the TMCO3 expression. Then, we found that TMCO3 could facilitate the membrane translocation and phosphorylation levels of AKT. Finally, our hypothesis was confirmed by targeting TMCO3 knockdown and inhibiting the AKT activity in animal experiments. This study demonstrates a new molecule that regulates AKT via the PI3K pathway, suggesting that TMCO3 can serve as a novel biomarker for HCC and potentially provide a therapeutic target for HCC treatment.

## 2 Results

### 2.1 TMCO3 Presents a High m^6^A Modification Level and High Expression Level in HCC

**Figure 1:**
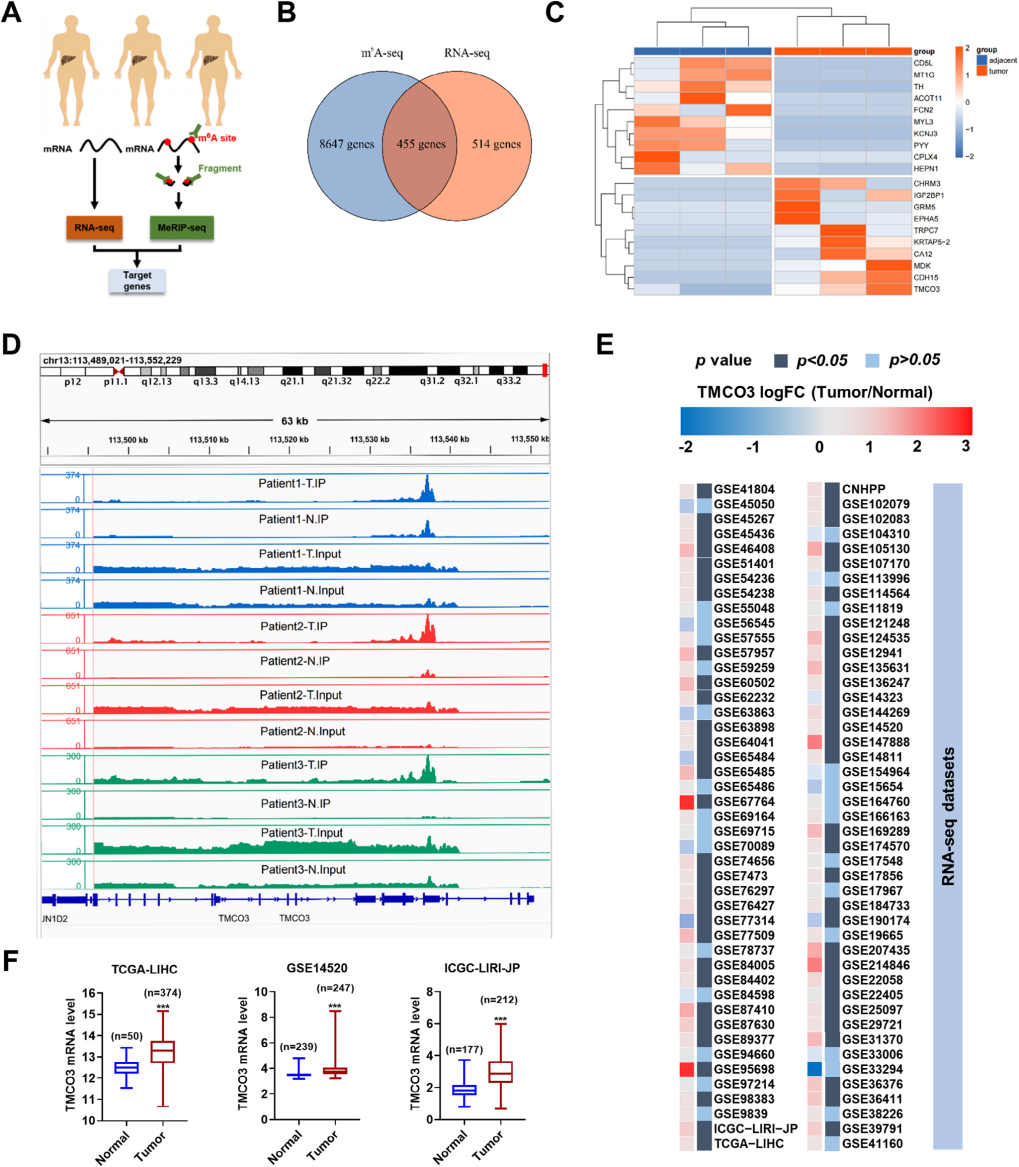
TMCO3 presents a high m^6^A modification level and high expression level in HCC. A) Schematics of MeRIP‐seq and RNA‐seq were performed on tumor and adjacent noncancerous tissues of three patients. B) Wayne diagram of gene intersections with statistical differences in MeRIP‐seq and RNA‐seq. C) The heat map shows the top 10 differentially expressed genes in gene intersections. D) m^6^A levels expression peak of TMCO3 in tumor and adjacent noncancerous tissues of three pairs of patients. E) RNA‐seq data from 92 GSE subsets showed the relative expression and *p*‐value of TMCO3 in tumor and adjacent noncancerous tissues. F) TMCO3 expression levels in tumor and adjacent noncancerous tissues in the three classic GSE subsets (TCGA‐LIHC, GSE14520, ICGC‐LIRI‐JP).

To identify potential targets that may be associated with m^6^A modification in HCC, three pairs of tumor and adjacent non‐cancerous tissues from HCC patients were collected (All three patients have a history of chronic viral hepatitis B, and two of them are accompanied by liver cirrhosis) and then both MeRIP‐seq and RNA‐seq were conducted (**Figure** **1**A). The overlap of up‐regulated genes in the results from both MeRIP‐seq and RNA‐seq was analyzed, revealing that 455 genes exhibited statistically significant differences (Figure 1B). Among these genes, we identified 10 genes that demonstrated higher expression in tumor tissues compared to adjacent non‐cancerous tissues, with TMCO3 ranking at the forefront (Figure 1C). It is noteworthy that TMCO3 has been seldom reported in relation to tumors, and even in the realm of other diseases, thus piquing our interest. Subsequently, pan‐cancer data from the TCGA database also revealed that in most cancer species, TMCO3 mRNA levels were significantly elevated in tumor tissues compared to adjacent non‐cancerous tissues (Figure S1A, Supporting Information). Next, we analyzed the abundance of m^6^A modification levels in TMCO3 across tumor and adjacent non‐cancerous tissues from three pairs of patients. The results suggested that the m^6^A peak level of TMCO3 in tumor tissues was significantly higher than that in adjacent non‐cancerous tissues, which matched our previous overlap analysis (Figure 1D). Then, we analyzed large‐scale data from 92 GSE datasets and observed significant alterations in TMCO3 mRNA levels in HCC tissues in 63 of these datasets (Figure 1E). Notably, in 57 out of these datasets, the TMCO3 mRNA level was significantly elevated in HCC tissues, and in three representative datasets: TCGA‐LIHC, GSE14520, and ICGC‐LIRI‐JP, TMCO3 mRNA levels were all increased in tumors (Figure 1F). Results from qRT‐PCR (quantitative reverse‐transcription PCR) and Western blots assay also showed that both TMCO3 mRNA and protein levels were significantly elevated in various HCC cell lines compared to normal liver cells (Figures S1B,C, Supporting Information). High TMCO3 expression was related to poor overall survival in TCGA‐LIHC and ICGC‐LIRI‐JP datasets (Figures S1D,E, Supporting Information).

These data suggested that TMCO3 showed a high m^6^A modification level and was highly expressed in HCC cell lines and HCC patients.

### 2.2 TMCO3 Promotes HCC Proliferation and Metastasis In Vivo and In Vitro

**Figure 2:**
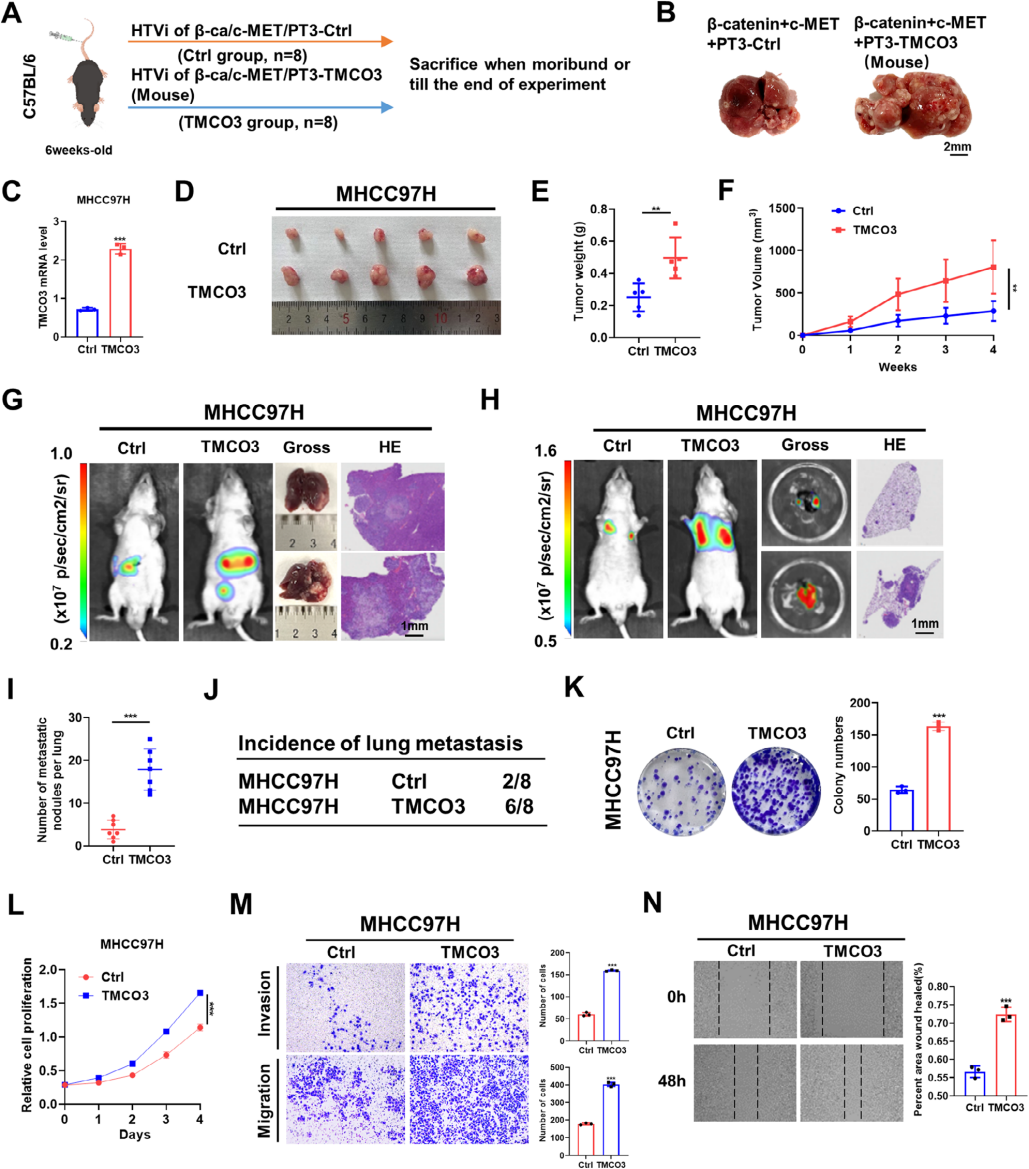
TMCO3 promotes HCC proliferation and metastasis in vivo and in vitro. A) Diagram of HTVi‐induced HCC models in C57BL/6 mice. B) The gross images of tumors in β‐catenin+c‐MET+PT3‐Ctrl group and β‐catenin+c‐MET+PT3‐TMCO3 (Mouse) group. C) The relative TMCO3 mRNA expression level in MHCC97H‐Ctrl group and MHCC97H‐TMCO3 group. D) The gross images of subcutaneous tumors in MHCC97H‐Ctrl and MHCC97H‐TMCO3 groups. E) The tumor weights of subcutaneous tumors in MHCC97H‐Ctrl and MHCC97H‐TMCO3 groups. F) The tumor volumes of subcutaneous tumors in MHCC97H‐Ctrl and MHCC97H‐TMCO3 groups. G) Representative images of the bioluminescence, gross, and HE staining of orthotopic xenograft tumors in MHCC97H‐Ctrl and MHCC97H‐TMCO3 groups. H) Representative images of the bioluminescence, gross, and HE staining of lung metastasis models in MHCC97H‐Ctrl and MHCC97H‐TMCO3 group. I) The statistical chart of metastatic nodules of the lung in MHCC97H‐Ctrl and MHCC97H‐TMCO3 groups. J) The schematic drawing of the incidence of lung metastasis in MHCC97H‐Ctrl and MHCC97H‐TMCO3 groups. K) Colony formation assays of MHCC97H cells in Ctrl and TMCO3 groups. L) CCK‐8 assays of MHCC97H cells in Ctrl and TMCO3 groups. M) Migration and invasion assays of MHCC97H cells in Ctrl and TMCO3 groups. N) Wound healing assays of MHCC97H cells in Ctrl and TMCO3 groups.

To delve deeper into the function of TMCO3 in HCC, we hydrodynamically co‐injected the c‐Met and N90‐β‐catenin constructs together with PT3‐Ctrl/TMCO3(Mouse) plasmids in C57BL/6 mice (**Figure** **2**A). Mice were closely observed and humanely euthanized upon developing substantial abdominal tumors and reaching a state of severe ill health. Interestingly, we observed that in the 8th week, mice in the PT3‐TMCO3(Mouse) group showed mortality, and these mice presented a more significant liver tumor burden compared to the control group (Figure 2B). This was accompanied by increased liver‐to‐body weight ratio and death rate in PT3‐TMCO3(Mouse) group (Figure S2A–C, Supporting Information). Subsequently, we constructed stable cell lines with MHCC97H overexpressing TMCO3 and HLF knockdown TMCO3 respectively, and verified their level of mRNA expression (Figure 2C; Figure S2D, Supporting Information). First, MHCC97H cells overexpressing TMCO3 were subcutaneously injected into nude mice. After four weeks, the group with TMCO3 overexpression showed larger tumor volume and increased tumor weight compared to the control group (Figure 2D–F). Then we injected HLF cells with TMCO3 knockdown in the same way, the results were opposite (Figure S2E–G, Supporting Information). Further to investigate the effect of TMCO3 on HCC metastasis, the MHCC97H cell line control and overexpressing TMCO3 groups were used to construct orthotopic xenograft models and nude mice tail vein models. After six weeks, the overexpressing TMCO3 group substantially enhanced the capacity of HCC cells to form secondary lesions in the liver compared to the control group (Figure 2G). In the nude mice tail vein models, the mice injected with TMCO3 overexpression cells showed more lesions and nodules compared to the control group after eight weeks (Figure 2H–J). In vitro experiments, colony formation assays and CCK‐8 assays indicated that overexpression of TMCO3 caused increased cell proliferation and colony numbers in MHCC97H cells, and knockdown TMCO3 in HLF cells resulted in the opposite results (Figure 2K,L; Figure S2H,I, Supporting Information). Then, the migration and invasion assays showed the increased ability of migration and invasion in MHCC97H overexpressing TMCO3 cells, and similar effects were observed in wound healing assays (Figure 2M,N). The opposite effects were found in HLF knockdown TMCO3 cells (Figure S2J,K, Supporting Information).

Altogether, our results showed TMCO3 could promote the progression and metastasis of HCC both in vivo and in vitro.

### 2.3 ALKBH5 Downregulates TMCO3 in an m^6^A‐Dependent Manner

**Figure 3:**
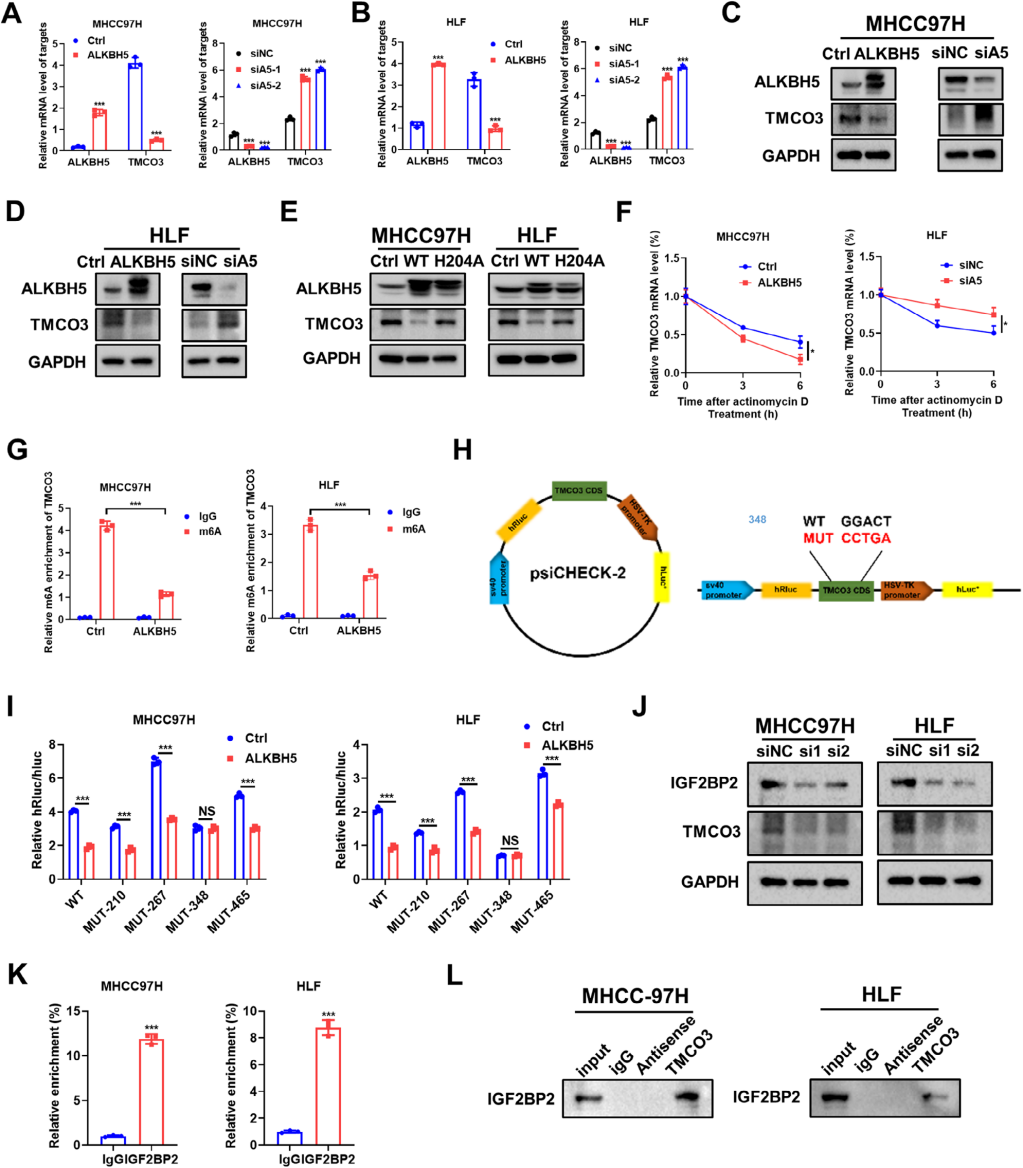
ALKBH5 downregulates TMCO3 in an m^6^A‐dependent manner. A) The relative ALKBH5 and TMCO3 mRNA levels of MHCC97H cells after ALKBH5 overexpression or knockdown. B) The relative ALKBH5 and TMCO3 mRNA expression levels of HLF cells after ALKBH5 overexpression or knockdown. C) The protein levels of ALKBH5 and TMCO3 in MHCC97H cells after ALKBH5 overexpression or knockdown. D) The protein levels of ALKBH5 and TMCO3 in HLF cells after ALKBH5 overexpression or knockdown. E) The protein levels of ALKBH5 and TMCO3 of HLF cells in Ctrl, ALKBH5‐WT, and ALKBH5‐H204A groups. F) The relative mRNA expression levels of TMCO3 after actinomycin D treatment in MHCC97H and HLF cells. G) MHCC97H and HLF cells were transfected with Ctrl or ALKBH5 plasmids and then subjected to MeRIP‐qPCR assays. H) The Schematic drawing of psiCHECK2‐TMCO3 plasmid and the potential m^6^A modified site. I) The relative luciferase activities of different mutations in TMCO3 CDS region after ALKBH5 expression in MHCC97 and HLF cells. J) The relative TMCO3 mRNA levels of MHCC97H and HLF cells after IGF2BP2 knockdown. K) RIP assays were carried out using anti‐IGF2BP2 antibody, and the RNA was extracted from protein G‐agarose with anti‐IGF2BP2 antibody, or with control IgG. Then qPCR assays were performed. L) The western blotting results of RNA‐pull down in MHCC97 and HLF cells.

Considering that we identified the molecule TMCO3 through MeRIP‐seq and RNA‐seq of HCC patient tumor and adjacent non‐cancerous tissues, existing m^6^A regulators were preliminary to explore what type of m^6^A regulator affects TMCO3. We detected the protein level of TMCO3 in MHCC97H cells after siRNA‐mediated knockdown of several common m^6^A regulators, including METTL3, METTL14, WTAP, FTO, and ALKBH5 (Figure S3A, Supporting Information). The western blot results indicated that only si‐ALKBH5 resulted in an elevation in the protein level of TMCO3, while the others showed no significant influences (**Figure** **3**C,D). RT‐qPCR results revealed that TMCO3 mRNA level was significantly upregulated upon ALKBH5 inhibition in both MHCC97H cells and HLF cells, whereas overexpressing of ALKBH5 resulted in downregulation of TMCO3 mRNA level (Figure 3A,B). In 293 T cells, exogenous plasmids of ALKBH5 and TMCO3 were co‐transferred. The western blots result also showed that the protein level of TMCO3 decreased with the increase of ALKBH5 concentration dose (Figure S3B, Supporting Information). Then we constructed psiCHECK2 plasmid containing TMCO3 wt region and conducted the dual luciferase reporter gene experiment in 293 T cells. The results showed that the activity of *Renilla* luciferase in the ALKBH5 overexpression group was significantly downregulated (Figure S3C, Supporting Information). Given that ALKBH5 plays a pivotal role as an “eraser” in m^6^A modifications, an inactive ALKBH5‐mut (mutant) was used to test whether ALKBH5‐mediated TMCO3 regulation was dependent on m^6^A modification. When ALKBH5‐mut was overexpressed in both MHCC97H and HLF cells, the protein level of TMCO3 was downregulated only in the ALKBH5‐wt group, not in the ALKBH5‐mut group (Figure 3E). Subsequent RNA decay assays demonstrated that the stability of TMCO3 mRNA significantly decreased upon overexpression of ALKBH5 in MHCC97H cells, while it increased following the knockdown of ALKBH5 in HLF cells (Figure 3F). Furthermore, m^6^A‐specific antibodies in the TMCO3 mRNA within both MHCC‐97H and HLF cells were significantly enriched (Figure 3G). Further to explore the specific sites of m^6^A modification on TMCO3, several potential sites on the CDS region of TMCO3 were predicted through the online prediction website SRAMP analysis (http://www.cuilab.cn/sramp). Based on the results, several potential m^6^A sites were identified with high or very high confidence (Figure S3C, Supporting Information). To confirm that these potential sites are critical for TMCO3 regulation, point mutations were introduced into putative m^6^A sites: MUT‐210, MUT‐267, MUT‐348, and MUT‐465 (Figure 3H). Subsequently, the dual luciferase reporter assays showed that ALKBH5 did not bind to TMCO3 when the MUT‐348 site was mutated in both MHCC97H cells and HLF cells (Figure 3I). Given that “readers” are directly involved in the m^6^A modification process, we next explore potential readers that may regulate TMCO3. RT‐qPCR results revealed that in MHCC97H cells, upon knocking down IGF2BP1, IGF2BP3, YTHDF1, YTHDF2, YTHDF3, eIF3, HNRNPC, HNRNPG, YTHDC1, and YTHDC2, the mRNA level of TMCO3 did not change to any significant extent (Figures S3D,E, Supporting Information). However, after knocking down IGF2BP2, the TMCO3 mRNA level and protein level were significantly decreased (Figure S3F,G, Supporting Information; Figure 3J). To further confirm that it was IGF2BP2 that regulated TMCO3 rather than other readers, we knocked down several readers that play a role in regulating mRNA translation in the MHCC97H cell line, such as IGF2BP1, IGF2BP3, YTHDF1, EIF3A, and YTHDC2. The western blots result showed that the knockdown of these several readers did not change the protein level of TMCO3 (Figure S3H, Supporting Information). Moreover, the RIP‐qPCR results confirmed that the TMCO3 RNA binds to IGF2BP2 in MHCC97H cells and HLF cells (Figure 3K). The RNA‐pull‐down assay was performed to confirm the physical interaction between TMCO3 and IGF2BP2 in MHCC97H cells and HLF cells (Figure 3L).

These results revealed that ALKBH5 downregulated TMCO3 in an m^6^A‐dependent manner, and this effect was mediated by IGF2BP2 recognition.

### 2.4 TMCO3 Binding with AKT Directly and Increases AKT Phosphorylation in a PI3K Dependent Manner

**Figure 4:**
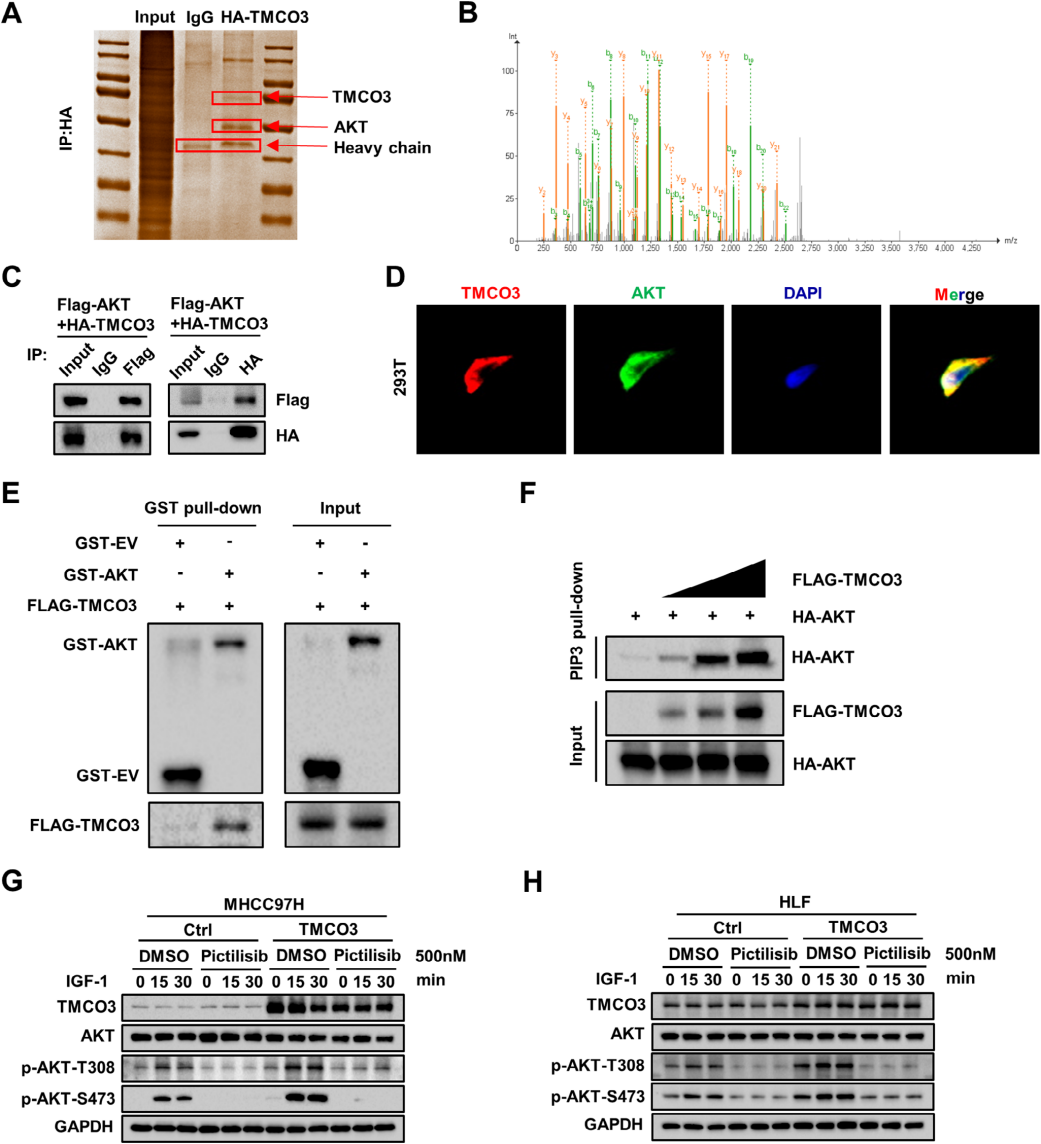
TMCO3 binding with AKT directly and increases AKT phosphorylation in a PI3K dependent manner. A) The silver staining diagram of IP. B) Secondary peptide distribution of mass spectrometry results. C) The interaction between TMCO3 and AKT was confirmed in 293 T cell line. D) Immunofluorescence showed that TMCO3 and AKT were colocalized in the cytoplasm. E) GST‐pull‐down assay showed the direct binding between TMCO3 and AKT. F) PIP_3_ pull‐down assay showed the interaction between AKT and PIP_3_ after transfecting TMCO3. G) MHCC97H cells with overexpression of TMCO3 or control were pre‐treated with Pictilisib or DMSO control for 2 h before IGF‐1 (100 nm) stimulation. Then western blots were performed. H) HLF cells with overexpression of TMCO3 or control were pre‐treated with Pictilisib or DMSO control for 2 h before IGF‐1 (100 nm) stimulation. Then western blots were performed.

TMCO3, a member of the monovalent cation: proton antiporter 2 (CPA2) transporter proteins family, which has been less studied in tumors. The oncogenic role of TMCO3 in HCC has been previously reported, but the specific mechanisms are still unclear.^[^ ^10^ ^]^ The further to explore the mechanism through which TMCO3 promotes the progression of HCC, immunoprecipitation (IP) followed by MS was performed to identify potential TMCO3 interaction proteins, AKT appeared at the top of the ranking (**Figure** **4**A,B; Table S4, Supporting Information). The interaction between TMCO3 and AKT was verified in 293 T cells, and then verified their co‐expression in the cytoplasm and membrane through immunofluorescence (IF) assays (Figure 4C,D). To ascertain whether TMCO3 could interact directly with AKT, we further constructed and purified GST‐AKT from *E.coli* and purified Flag‐TMCO3 from 293 T cells, then GST pull‐down assays were performed (Figure 4E). The results showed that TMCO3 could bind to AKT directly. Given the recruitment of AKT to the cell membrane and binding to PIP_3_ is the initial step of AKT activation. To assess test whether TMCO3 could promote the binding of AKT and PIP_3_, the PIP_3_ pull‐down assays showed that the binding of AKT and PIP_3_ was gradually increased in a TMCO3 dose‐dependent manner (Figure 4F). Moreover, to observe whether TMCO3 could bind to PIP_3_, we conducted a lipid strip assay. The results indicated that, in contrast to the positive binding of GST‐AKT to both PIP_2_ and PIP_3_, FLAG‐TMCO3 did not exhibit binding to PIP_3_. (Figure S4‐1A,B, Supporting Information).

Next, we tested whether TMCO3 has an effect on downstream signaling in PI3K‐AKT pathway. RNA sequencing was performed in TMCO3‐overexpression and control cells. Kyoto Encyclopedia of Genes and Genomes (KEGG) pathway enrichment analysis and Gene Set Enrichment Analysis (GSEA) indicated that genes regulated by TMCO3‐overexpression exhibited the greatest enrichment in the PI3K‐AKT pathway (Figure S4‐1C,D, Supporting Information). Further to investigate whether TMCO3 affects the expression and activation levels of the AKT protein, we assessed the protein expression levels of AKT, p‐AKT‐T308, and p‐AKT‐S473 in MHCC97H and HLF cell lines with stably overexpressed and knocked‐down TMCO3. Western blot results indicated that the expression levels of AKT protein remained unaltered with either overexpression or knockdown of TMCO3. However, the p‐AKT‐T308 and p‐AKT‐S473 levels were increased with TMCO3 overexpression and decreased upon TMCO3 knockdown (Figure S4‐1E,F, Supporting Information). Meanwhile, we carried out CO‐IP experiments on TMCO3 with some classic PI3K subunits and other classic molecules that can affect AKT activation (classic regulatory subunits and other proteins: P110α, P110α, p85α, PDK1, PHLPP2, and SIN1). Unfortunately, the results showed that these proteins seem didn't bind to TMCO3 (Figure S4‐1G, Supporting Information). Next, we also explored whether TMCO3's promotion of AKT activation depends on its function as an antiporter. We added Dimethylamiloride (which is an antiporter inhibitor) to the cell lines overexpressing TMCO3 in MHCC97H and HLF respectively. The western blot results showed that compared with the group only overexpressing TMCO3, the AKT activation level in the group with Dimethylamiloride added didn't seem to change (Figure S4–2A, Supporting Information). Therefore, we speculate that TMCO3 may not affect the AKT activation level through its antiporter function. Subsequently, we investigated whether the regulation of AKT phosphorylation by TMCO3 was dependent on the demethylation modification of ALKBH5 through western blots assay.^[^ ^11^ ^]^ The results demonstrated that upon transfection with ALKBH5‐wt, the protein level of TMCO3 was downregulated, and the phosphorylation levels at the T308 and S473 sites of AKT were significantly reduced compared to the control group. When the ALKBH5‐mut was transfected, the protein level of TMCO3 showed a notable recovery compared to the wild‐type ALKBH5 transfection group, and the phosphorylation at the T308 and S473 sites of AKT was also partially restored. These findings suggest that the regulation of AKT phosphorylation levels by TMCO3 was contingent upon the demethylase activity of ALKBH5 (Figure S4‐2B, Supporting Information).

IP assays then showed that the interaction between TMCO3 and AKT was enhanced with increasing IGF stimulation time and the dosage of TMCO3, respectively (Figure S4‐2C,D, Supporting Information). Since IGF is a major cytokine that stimulates the activation of the PI3K‐AKT pathway, we speculated whether TMCO3 is dependent on the activation of PI3K pathway to increase the phosphorylation level of AKT. To verify this hypothesis, we treated MHCC97H and HLF cells with PI3K inhibitor Pictilisib, or DMSO control. In the Pictilisib treatment group, overexpression of TMCO3 did not affect p‐AKT‐T308 and p‐AKT‐S473 levels (Figure 4G,H). This result confirmed that AKT activation by TMCO3 is contingent upon the activation of the PI3K pathway. Subsequently, we performed IP assays in MHCC97H and HLF cells. The results demonstrated that the binding between TMCO3 and AKT was significantly enhanced under IGF stimulation, but this effect was obviously weakened after the treatment of Pictilisib (Figure S4‐2E,F, Supporting Information).

In summary, these results indicated that TMCO3 directly binds to AKT and activates AKT in a PI3K‐dependent manner.

### 2.5 The IGF‐1‐Stimulated Phosphorylation on the Serine 85 Site of TMCO3 Facilitates the Membrane Localization and Activation of AKT

**Figure 5:**
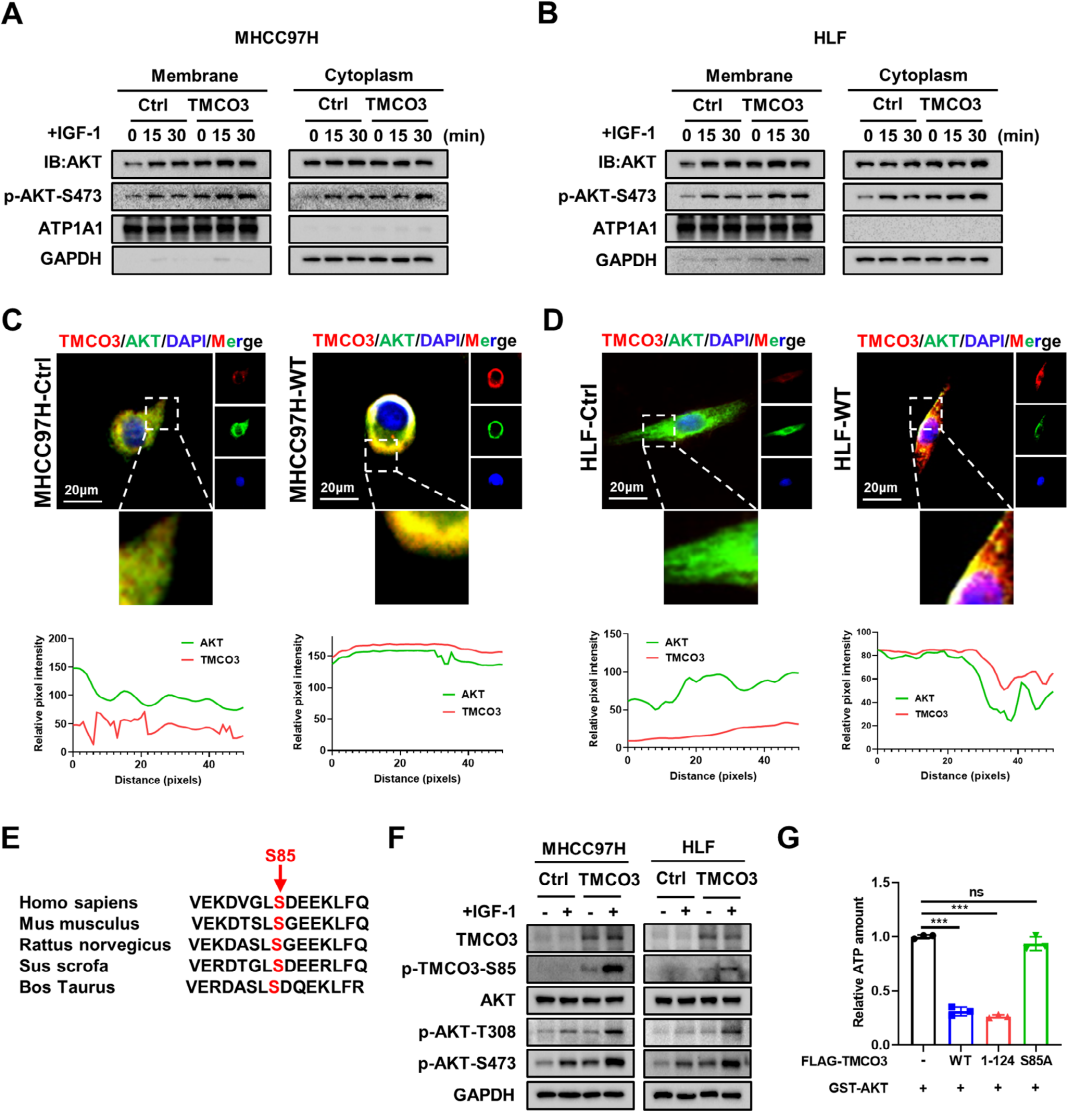
The IGF‐1‐stimulated phosphorylation on the serine 85 site of TMCO3 facilitates the membrane localization and activation of AKT. A) The Cytoplasm and membrane protein fractionation assay was performed in MHCC97H cells after overexpression of TMCO3 or control. B) The Cytoplasm and membrane protein fractionation assay was performed in HLF cells after overexpression of TMCO3 or control. C) Immunofluorescence images showed the location of AKT in MHCC97H cell after overexpression of TMCO3. D) Immunofluorescence images showed the location of AKT in HLF cell after overexpression of TMCO3. E) Schematic diagram of TMCO3 85th amino acid in different species. F) The western blot results showed the protein levels of p‐TMCO3‐S85 and other targets in MHCC97H and HLF cells after IGF‐1 stimulation. G) The chemiluminescent kinase activity assay reflected the differences in the residual ATP levels among the various groups.

As a multi‐transmembrane protein, TMCO3 is predominantly localized in the plasma membrane and cytoplasm. A prerequisite for AKT activation is its transfer from cytoplasm to the inner surface of plasma membrane first, followed by its binding to PIP_3_, which in turn activates the T308 site by PDK1.^[^ ^12^ ^]^ We thus hypothesized that TMCO3 might increase the membrane location of AKT. Through cytoplasm‐membrane protein fractionation assays in MHCC97H and HLF cells, overexpression of TMCO3 was found to significantly enhance the membrane localization of AKT during stimulation of IGF‐1, while TMCO3 knockdown had the opposite effect (**Figure** **5**A,B; Figure S5A,B, Supporting Information). Consistently, our IF experiments confirmed this hypothesis, demonstrating that overexpression of TMCO3 could indeed promote the membrane localization of AKT (Figure 5C,D). Based on previous studies, little is known about the function of TMCO3 as a membrane protein.^[^ ^13^ ^]^ To further explore the specific region of TMCO3 that interacts with AKT, we constructed truncated plasmids of TMCO3, namely TMCO3(1‐124), TMCO3(1‐204), TMCO3(204‐677), and TMCO3(124‐677), based on the functional domain distribution of TMCO3. These constructs were co‐transfected with AKT in 293T cells. IP results revealed that AKT binds only to TMCO3(1‐124), TMCO3(1‐204), and the full‐length TMCO3, indicating that the TMCO3(1‐124) region may be responsible for the interaction between TMCO3 and AKT (Figure S5C,D, Supporting Information). Therefore, we further hypothesized whether TMCO3 itself possessed phosphorylation sites that could be activated, thus playing a kinase receptor‐like role in triggering AKT phosphorylation. To examine this hypothesis, we used the Cell Signaling Technology (CST) MS database (https://www.phosphosite.org) to predict potential phosphorylation residues in the TMCO3 CDS domain (Figure S5E, Supporting Information). Further to determine which residue was the requisite target, we generated the phosphor‐deficient mutants for TMCO3 (S to A) and observed that TMCO3‐S85A, not S134A or S638A, could markedly decrease p‐AKT‐T308 and p‐AKT‐S473 levels in MHCC97H cells (Figure S5G, Supporting Information). Subsequently, the co‐IP data revealed that compared with the empty vector control, transfection of TMCO3‐S85A reduced serine/threonine phosphorylation levels of AKT, as detected by immunoblotting with phospho‐Thr/Ser–specific antibodies (Figure S5H, Supporting Information). Then, we selected homologous sequences from various species sources in the Uniprot database. After aligning them using Clustal Omega, we intercepted the sequence interval of 1–124, the amino acid sequence of the 1–124 segment of TMCO3 is highly conserved. Moreover, the 85th serine site is also highly conserved across different species, which also indirectly indicates that this region is most likely the main region for activating AKT (Figure 5E; Figure S5E, Supporting Information). To further confirm that the 85th serine is the specific site of phosphorylation on TMCO3, we generated a site‐specific antibody that recognizes the phosphorylated S85 site on TMCO3. The western blots results demonstrated that, following the addition of IGF‐1 stimulation, overexpression of TMCO3 led to a significant increase in p‐TMCO3‐S85 level, and it resulted in a notable enhancement in p‐AKT‐T308 and p‐AKT‐S473 levels (Figure 5F). In addition, we conducted kinase activity assays to observe differences in kinase activity among various groups by comparing the residual amounts of ATP after enzymatic reactions in each group. The results indicated that, compared to the control group, the TMCO3‐wt group exhibited a significant decrease in the residual ATP levels. The TMCO3(1‐124) group showed a trend consistent with that of the TMCO3‐wt group. However, when the 85th serine site was mutated, the residual ATP levels showed no significant difference compared to the control group (Figure 5G). Next, the TMCO3 knockout MHCC97H cells were constructed, and western blot results revealed that p‐TMCO3‐S85 level was virtually undetectable after the knockout of TMCO3. The p‐TMCO3‐S85 level in the TMCO3 knockout cell lines modestly increased following the TMCO3‐S85A restoration, while it significantly rose after TMCO3‐S85D was restored. The p‐AKT‐S473 level mirrored the trend seen with p‐TMCO3‐S85 (Figure S5I, Supporting Information). Hence, these findings implied that IGF‐1 mediates the activation of phosphorylation on the 85th serine site of TMCO3, which in turn, facilitates the membrane localization and activation of AKT.

### 2.6 Mutation on the Serine 85 of TMCO3 Inhibits the Membrane Location and Kinase Activity of AKT, Ultimately Impeding HCC Progression

**Figure 6:**
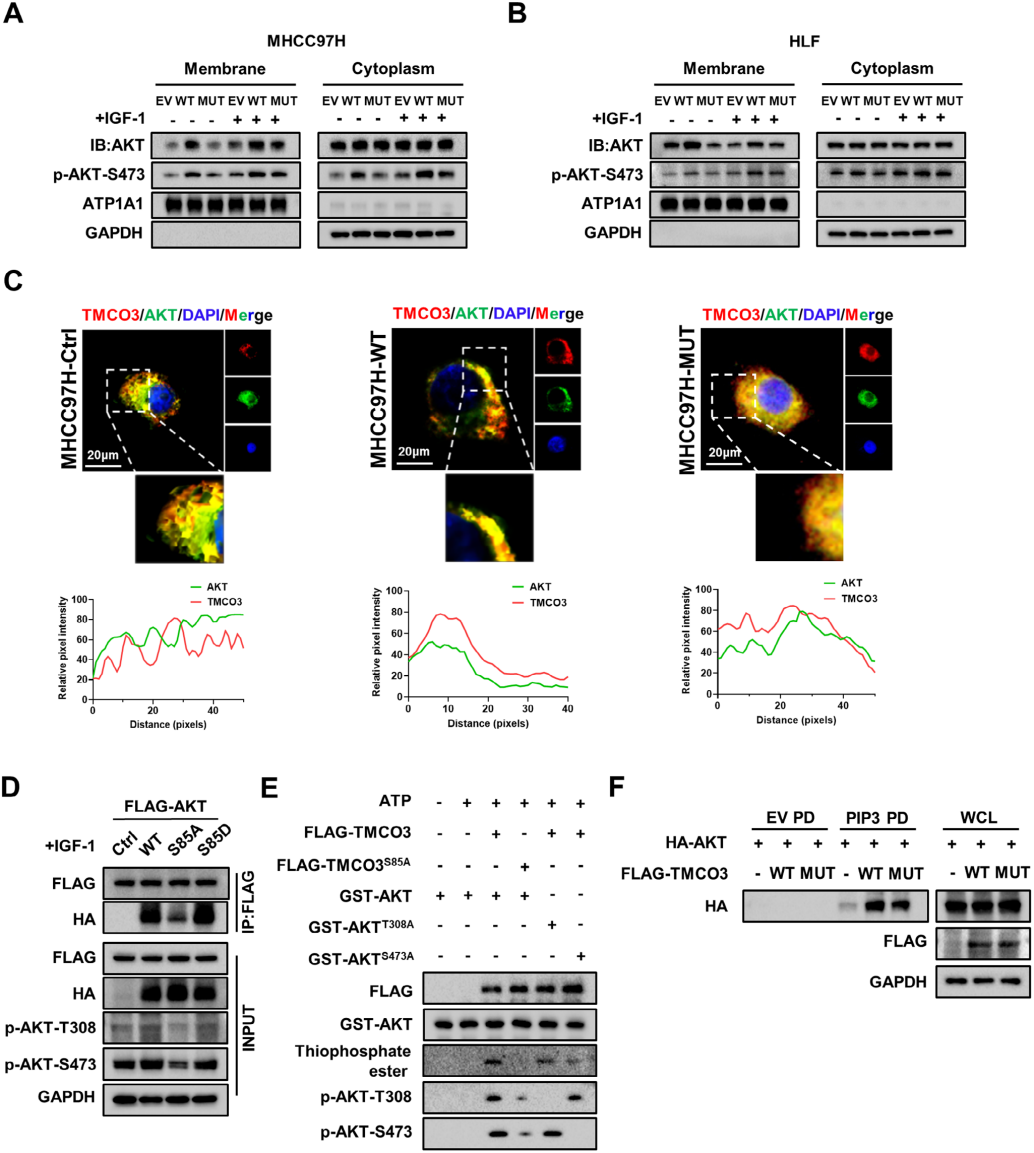
Mutation on the Serine 85 of TMCO3 inhibits AKT membrane location and kinase activity, ultimately impeding HCC progression. A) The Cytoplasm and membrane protein fractionation assay was performed in MHCC97H cells after overexpression of TMCO3‐WT, TMCO3‐MUT (S85A), or control. B) The Cytoplasm and membrane protein fractionation assay was performed in HLF cells after overexpression of TMCO3‐WT, TMCO3‐MUT (S85A), or control. C) Immunofluorescence images showed the location of AKT in MHCC97H cell after overexpression of TMCO3‐WT, TMCO3‐MUT (S85A), or control. D) IP assays showed the interaction between TMCO3 and AKT after transfection of TMCO3‐WT, TMCO3‐S85A, TMCO3‐S85D, or control. E) Western blots on the products from in vitro kinase assays assessing purified FLAG‐TMCO3/ TMCO3^S85A^ phosphorylation of GST‐AKT/AKT^T308A^/AKT^S473A^. F) PIP_3_ pull‐down assay showed the interaction between AKT and PIP_3_ after transfection of TMCO3‐WT and TMCO3‐S85A.

Previous data verified that phosphorylation on the S85 site of TMCO3 could promote the membrane localization and activation of AKT. Then, we investigated whether mutating S85 site could alleviate this effect. Through cytoplasm‐membrane protein fractionation assays and IF assays, our results highlighted that compared to the TMCO3‐WT group, the distribution of AKT within the cell membrane of the TMCO3‐S85A group was significantly reduced, with a corresponding significant decrease in the p‐AKT‐S473 level (**Figure** **6**A–C; Figure S6A, Supporting Information). Next, whether mutations on S85 site could weaken the binding of TMCO3 and AKT was ascertained. Co‐IP results showed that compared with TMCO3‐WT, the binding of TMCO3‐S85A to AKT was significantly weakened, while the binding of TMCO3‐S85D to AKT was restored (Figure 6D). Then, to ascertain whether TMCO3 could directly phosphorylate both the T308 and S473 sites of AKT simultaneously, we conducted in vitro kinase assays.^[^ ^14^ ^]^ The results showed that when the S85 site of TMCO3 was mutated, the phosphorylation levels at T308 and S473 sites of the wild‐type AKT purified protein were down‐regulated to varying degrees. When the T308 site of AKT was mutated, the activating effect of TMCO3 on GST‐AKTT^308A^ almost completely disappeared. And when the S473 site of AKT was mutated, the activating effect of TMCO3 on GST‐AKT^S473A^ also nearly vanished. Combining with the results of the Thi‐ester phosphorylation antibody, we concluded that TMCO3 could directly phosphorylated the T308 and S473 sites of AKT, and this activating effect may be more significant for the phosphorylation of the S473 site (Figure 6E). The PIP_3_ pull‐down assays also showed that following the mutation on site 85 of TMCO3, the binding of PIP_3_ to AKT was notably weakened compared to TMCO3‐WT group, which was consistent with the previous results (Figure 6F). The better to evaluate the effect of mutation on S85 site, TMCO3‐WT, TMCO3‐S85A, TMCO3‐S85D, TMCO3‐S134A, and TMCO3‐S638A in MHCC97H‐KO‐TMCO3 cell lines were restored.^[^ ^15^ ^]^ CCK‐8 assays and colony formation assays demonstrated that compared to KO+WT group, the cell proliferation capacity of KO+S85A group was significantly reduced (Figure S6B,C, Supporting Information). The other groups showed similar effects to those of KO+WT group. Subsequently, the subcutaneous tumor injection model demonstrated findings consistent with in vitro experiments. Tumor weight and volume were significantly increased in the KO+WT group as compared to the KO group. However, this effect almost vanished after TMCO3‐S85A restoration, and the other groups exhibited trends similar to the KO+WT group (Figure S6D–F, Supporting Information). Collectively, these results support mutation of S85 site on TMCO3 impairs AKT membrane location and kinase activity, finally negatively regulating oncogenic functions of HCC.

### 2.7 Combined Treatment with AAV‐shTMCO3 and MK2206 Dramatically Decreases AKT Phosphorylation and Inhibits Tumorigenesis in Mouse Model

**Figure 7:**
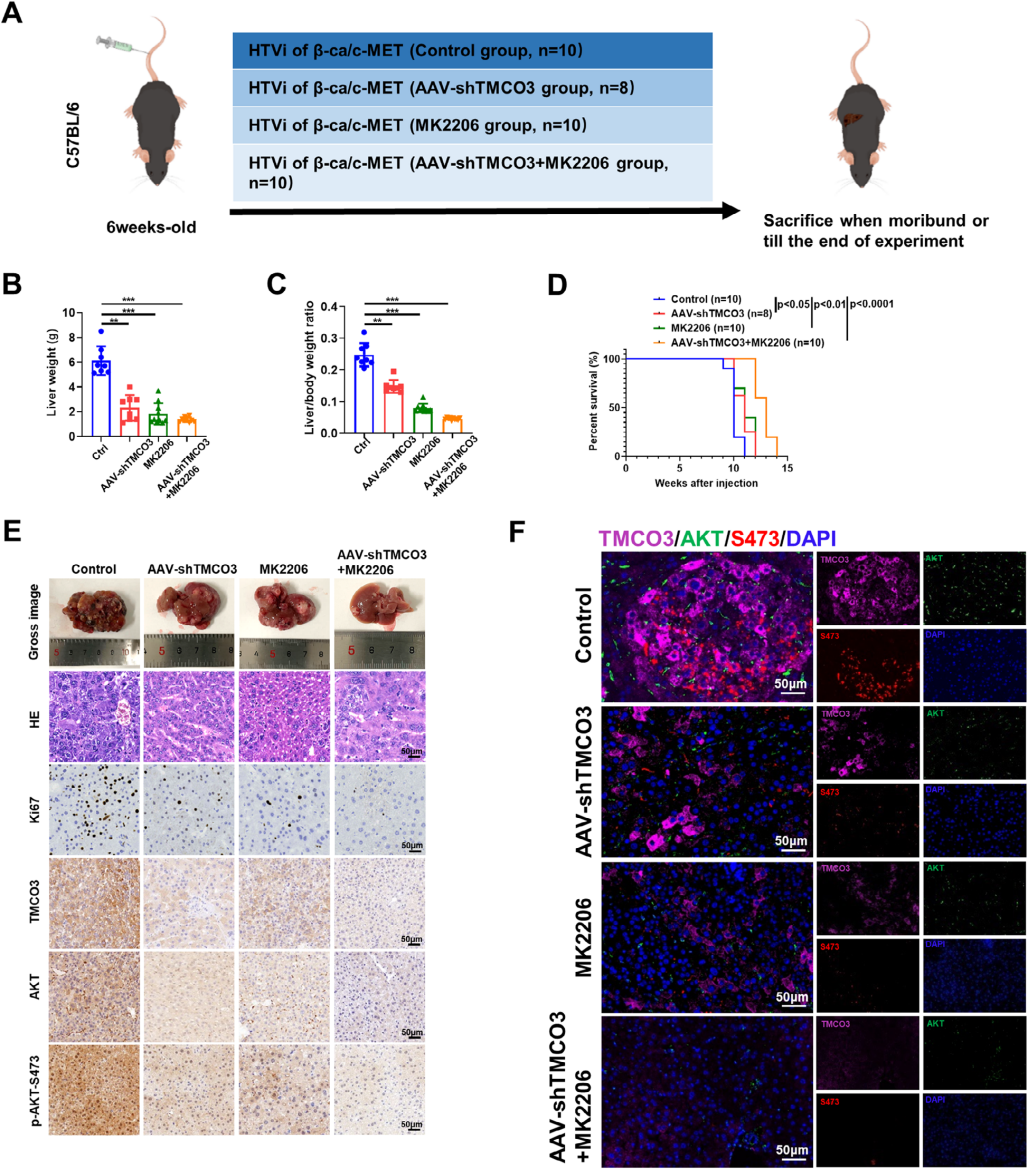
Combined treatment with AAV‐shTMCO3 and MK2206 dramatically decreases AKT phosphorylation and inhibits tumorigenesis in mouse models. A) The schematic diagram of HTVi‐induced HCC models in C57BL/6 mice and treatment groups. B) The liver weight statistics chart in four different groups. C) The statistics chart of liver/body weight ratio in four groups. D) The survival rates of mice in four groups. E) The gross images, HE staining, and IHC staining of four groups. F) The mIHC images include relative targets in four groups.

Next, to investigate whether targeting TMCO3 could augment the therapeutic effectiveness of MK2206, an allosteric AKT inhibitor, a hydrodynamic model was established for c‐Met/N90‐β‐catenin ‐driving hepatocarcinogenesis (**Figure** **7**A).^[^ ^16^ ^]^ Initially, the knockdown efficiency of TMCO3 (mouse) in Hep1‐6 cells was demonstrated (Figure S7‐1A, Supporting Information).^[^ ^17^ ^]^ Following this, we utilized adeno‐associated virus 8 (AAV8) to package the short hairpin RNA (shRNA) targeting TMCO3 and designed a control vector for AAV8 as a measure to verify the success of the AAV8 injection (Figure S7‐1B, Supporting Information).^[^ ^18^ ^]^ The results showed that compared to the control group, both the AAV‐shTMCO3 group and the MK2206 treatment group had significantly lower tumor burdens.^[^ ^19^ ^]^ The combined treatment of AAV‐shTMCO3 and MK2206 resulted in the most substantial reduction in tumor burden and also exhibited the best survival rate (Figure 7B–E). Overexpression of c‐Met, N90‐β‐catenin, and knockdown of TMCO3 were validated by western blots in these four groups (Figure S7‐1C, Supporting Information). The results of immunohistochemical (IHC) staining also indicated that the expression levels of KI67, TMCO3, and p‐AKT‐S473 were significantly downregulated in both the AAV8 and MK2206 groups, and the most pronounced downregulation was observed in the combined treatment group (Figure 7E). Simultaneously, multiplex immunohistochemistry (m‐IHC) results also revealed that the decrease in the levels of TMCO3 and p‐AKT‐S473, as well as the reduction in AKT membrane localization, were most significant in the combined treatment group compared to the control group (Figure 7F; Figure S7‐1D,E, Supporting Information). Then, we used the MHCC97H cell line to construct orthotopic xenograft models in nude mice and divided these mice into four groups as before. The results mirrored our previous findings, where the combined treatment group had the lightest tumor burden and the fewest intrahepatic metastatic foci (Figure S7‐2A–D, Supporting Information). Western blots and IHC staining results validated the knockdown efficiency of TMCO3, the expression level of 473, and the localization of AKT (Figure S7‐2E,F, Supporting Information). In conclusion, these data suggested that targeting TMCO3 combined with MK2206 could significantly decrease AKT phosphorylation and inhibit the progression of HCC.

### 2.8 The Levels of TMCO3 and p‐TMCO3‐S85 are Positively Correlated with HCC and Predict Poor Prognosis

**Figure 8:**
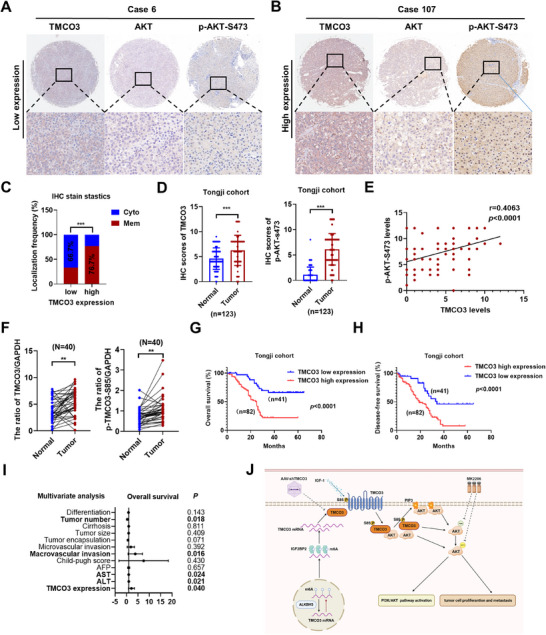
The levels of TMCO3 and p‐TMCO3‐S85 are positively correlated with HCC and predict poor prognosis. A) The IHC staining of case 6 of TMCO3, AKT, P‐AKT‐S473 targets in tissue microarrays. B) The IHC staining of case 107 of TMCO3, AKT, and P‐AKT‐S473 targets in tissue microarrays. C) Statistical chart showed the cytoplasmic and membrane localization of AKT in groups with high or low TMCO3 expression. D) The statistical chart showed the TMCO3 IHC scores in adjacent noncancerous or tumor tissues of HCC patients in the Tongji cohort. E) Pearson's correlation analysis of the TMCO3 and P‐AKT‐S473 IHC scores in tissue microarrays. F) The Statistical chart of TMCO3 and P‐TMCO3‐S85 protein levels in adjacent noncancerous or tumor tissues of 40 pairs of HCC patients. G) The overall survival with TMCO3 low expression or TMCO3 high expression of 123 pairs of HCC patients in the Tongji cohort. H) The disease‐free survival with TMCO3 low expression or TMCO3 high expression of 123 pairs of HCC patients in the Tongji cohort. I) Forest plot of the multivariate analysis for overall survival. J) Schematic model of the mechanism underlying TMCO3‐drived HCC progression and relative treatment.

The previous data validated the influence of TMCO3 on AKT and its inhibitory role in HCC progression through both cell and animal experiments. Next, to observe the expression of TMCO3 and its associated prognosis in a cohort of patients with HCC from Tongji Hospital, we selected tissue microarrays from 123 pairs of HCC patient tumors and adjacent non‐cancerous tissues for IHC staining of TMCO3, AKT, and P‐AKT‐S473 (**Figure** **8**A,B; Table S1, Supporting Information). The results showed that the expression levels of TMCO3 and P‐AKT‐S473 in cancer tissues were significantly higher than those in adjacent non‐cancerous tissues (Figure 8D). Moreover, in samples with a high TMCO3 expression, the proportion of AKT localized to the membrane was also notably higher than in samples with a low TMCO3 expression (Figure 8C). The Pearson correlation test indicated a significant positive correlation between the expression levels of P‐AKT‐S473 and TMCO3 (Figure 8E). Subsequently, western blotting was conducted to assess the expression levels of TMCO3 and p‐TMCO3‐S85 in 40 pairs of tumor and adjacent non‐cancerous tissues from HCC patients. The results revealed that both TMCO3 and p‐TMCO3‐S85 were significantly increased in cancer tissues compared to adjacent noncancerous tissues (Figure 8F; Figure S8A,B, Supporting Information). Consistent with the analysis of the TCGA database, the group with a high TMCO3 expression in the Tongji HCC patient cohort exhibited poorer overall survival and disease‐free survival rates (Figure 8G,H). Cox's multivariate analysis indicated that higher TMCO3 expression was an independent risk factor for shorter overall survival (Figure 8I; Table S2, Supporting Information). In summary, these data demonstrated that the levels of TMCO3 and p‐TMCO3‐S85 were highly expressed in HCC patients, and also correlated with worse OS and DFS in the Tongji HCC patient cohort.

## 3 Discussion

Nowadays, the surgical operations and medicine treatment for HCC worldwide have reached a certain bottleneck, with advanced HCC and some recurrent and metastatic HCC still associated with high mortality rates. Therefore, an urgent understanding of the molecular mechanisms underlying HCC progression and therapeutic strategies is needed. This study identified TMCO3, a highly expressed protein in HCC, through MeRIP‐seq and RNA‐seq of patient tumor tissues. Mechanically, ALKBH5 downregulates the m^6^A modification of TMCO3 mRNA, and IGF2BP2 binds to the m^6^A site of TMCO3 mRNA, maintaining its stability. TMCO3 promotes HCC progression by facilitating the membrane localization of AKT and increasing AKT activation. Significantly, targeting TMCO3 combined with MK2206, a classic allosteric inhibitor of AKT, effectively inhibits HCC progression.^[^ ^20^ ^]^

In recent years, studies on m^6^A modification in tumors have been emerging, with a growing number of reports focusing on its role in HCC.^[^ ^21^ ^]^ Classic m^6^A modifications are primarily conducted by “writers” such as METTL3/METTL14/WTAP or “erasers” like ALKBH5/FTO, modifying the m^6^A sites on targeted gene mRNA; “readers” such as IGF2BP1/2/3, YTHDF1/2/3 recognize and bind to the target gene mRNA for stabilization or degradation.^[^ ^22^ ^]^ Our study discovered for the first time that ALKBH5 downregulated the m^6^A methylation level of TMCO3 mRNA, with IGF2BP2 participating in its mRNA stabilization. Moreover, the specific site of m^6^A modification on TMCO3 mRNA was identified, which bears significant importance for our subsequent research.

The PI3K/AKT signaling pathway is participated in the progression of various tumors and is also highly activated in HCC.^[^ ^23^ ^]^ The sublocalization of AKT in tumor cells, and its activation of phosphorylation, can determine the role of the pathway in the tumor.^[^ ^24^ ^]^ In the current mainstream studies, the T308 site activation of AKT is primarily induced by PDK1, the S473 site is stimulated by mTORC2, and the influence of PTEN on inhibiting T308 site activation.^[^ ^25^ ^]^ In addition to these classic ways of activating AKT, there are also some non‐classic proteins that activate AKT independently of kinase function. For instance, the proto‐oncogene TCL1 can directly bind to AKT to promote its oligomerization, thereby altering the protein spatial conformation of AKT and facilitating its phosphorylation.^[^ ^26^ ^]^ Moreover, upon stimulation by IL – 1β, TRAF6 can undergo oligomerization, followed by K63‐linked polyubiquitination. This polyubiquitination does not lead to the degradation of TAK1, an interacting protein of TRAF6, via the proteasome pathway. Instead, it changes the activity state of TAK1. It is precisely due to the autoubiquitination of TRAF6 that the spatial conformation of the TRAF6‐TAK1 complex is altered, resulting in the activation of TAK1.^[^ ^27^ ^]^ In our study, we identified TMCO3, an oncogenic molecule, from MeRIP‐seq and RNA‐seq and confirmed its oncogenic effect. Subsequent mass spectrometry results revealed that AKT is a major candidate among binding proteins. Hence, we validated the direct bind between TMCO3 and AKT, discovering that TMCO3 binds with AKT more effectively when the PI3K pathway is activated. Furthermore, considering the cytoplasm and membrane localization of TMCO3, TMCO3 was found to facilitate the accumulation of AKT from the cytoplasm to the membrane and promote the phosphorylation level of AKT in HCC cell lines. Intriguingly, we speculated whether TMCO3 could function in a manner akin to that of other kinases to activate AKT and verified this hypothesis through phosphorylation mass spectrometry. We identified the 85^th^ serine site on TMCO3 and observed that the addition of IGF‐1 stimulation significantly increased the levels of p‐TMCO3‐S85. Subsequently, we mutated the 85^th^ serine site of TMCO3 from serine to alanine, noticing that TMCO3‐S85A largely abolished the function of promoting the membrane localization of AKT and activating AKT phosphorylation. This finding not only further elucidates the role of TMCO3 in HCC but also largely provides a new regulatory factor for the PI3K/AKT pathway, paving the way for future studies.

Further to highlight the significance of TMCO3 in HCC, we established a hydrodynamic‐induced HCC model in C57BL/6 mice and targeted TMCO3 via AAV‐shTMCO3, combined with MK2206 to observe the therapeutic effect on HCC. The results showed that both the AAV‐shTMCO3 group and the MK2206 group had certain therapeutic effects, whereas the combined treatment group exhibited the most significant effect, with the levels of p‐AKT‐T308 and p‐AKT‐S473 most noticeably reduced. Subsequently, we constructed an orthotopic xenograft model in nude mice and performed similar grouping, yielding consistent results. These findings suggest that TMCO3 exerts its corresponding function in vitro, and its influence on HCC in vivo has been further validated.

Next, we performed TMCO3, AKT, and p‐AKT‐S473 staining on tissue microarrays of 123 pairs of patients. The results revealed that the cell membrane localization of AKT in the group with a high TMCO3 expression was significantly higher than that in the group with a low TMCO3 expression. Moreover, the expression level of TMCO3 was positively correlated with p‐AKT‐S473. Subsequently, the protein expression of TMCO3 and p‐TMCO3‐S85 in patient tissues was detected. Their levels of expression were found to be significantly elevated in tumor tissues. Within our patient cohort, high TMCO3 expression also indicated poor overall survival and recurrence rates.

Overall, our study shows that TMCO3, a highly expressed and high m^6^A‐modification level molecule in HCC, is involved in the regulation of AKT through the PI3K/AKT pathway and ultimately promotes the progression of HCC (Figure 8J). These findings suggest that TMCO3 serves as a useful biomarker in HCC, and targeting TMCO3 may provide a potential therapeutic strategy for HCC, especially in HCC patients with a highly activated PI3K/AKT pathway.

## 4 Experimental Section

### Cell Culture and Reagents

THLE‐3, MHCC97H, Hep3B, HLF, HepG2, Alex, and LM3 cell lines were seeded in DMEM supplemented with 10% fetal bovine serum (FBS) (Tsingmu, Wuhan, China) at 37 °C in 5% CO2. The siRNAs were designed and provided by AuGCT Biotecho and the Lipomaster 3000 Transfection Reagent was obtained from Vazyme (Nanjing, China). The optical MEM was purchased from ThermoFisher (31985070).

### Animals Study

Male C57BL/6J mice and BALB/c nude mice were obtained from GemPharmatech (Nanjing, China) and were maintained in a Specific Pathogen Free (SPF) facility. For subcutaneous tumor assay in BALB/c nude mice, 1 × 10^6^ cells were diluted in 100 µL serum‐free medium and then injected in both armpits. For orthotopic xenograft models, 1 × 10^6^ cells were diluted in 30 µL serum‐free medium and then injected into the liver. For tail vein injection models, 1 × 10^6^ cells were diluted in 100 µL serum‐free medium and then injected in the tail vein. For the hydrodynamic tail vein injection (HTVi) induced HCC, 5–6 weeks male C57BL/6J mice were used. For each mouse, PT3‐c‐Met (20 µg)/PT3‐△N90‐β‐catenin (20 µg)/PT3‐Vector/TMCO3 (20 µg) and SB transposase plasmids (1/25 of the total plasmid mass) were dissolved in 2 mL PBS. The other details of animal studies were provided in the Supplementary Information.

### Statistical Analysis

Each value was shown as the average plus or minus the standard deviation. To assess statistical significance between two groups, Student's *t*‐tests (for normal distribution) or Wilcoxon signed‐rank tests (for matched pairs) were utilized. For multiple groups, statistical analysis was performed using either one‐way ANOVA or two‐way ANOVA. The Kaplan–Meier method illustrated the survival curves, while the log‐rank test evaluated their statistical significance. Correlations were evaluated using a Pearson correlation test. Appropriate statistical tests were conducted for all Figures, with *p* < 0.05 considered statistically significant: *p < 0.05; **p < 0.01; ***p < 0.001; ns: not significant. All statistical values were calculated using GraphPad Prism 8.0 software.

Other materials and methods are presented in the Supplementary Information.

### Ethics Approval and Consent to Participate

The ethics committee of Tongji Hospital, affiliated with Tongji Medical College at Huazhong University of Science and Technology (Wuhan, China) granted approval for the study. All research was conducted in accordance with both the Declarations of Helsinki and Istanbul. Written consent was given in writing by all subjects (TJ‐IRB20211214). All animal experimental procedures were also approved by the Committee on the Ethics of Animal Experiments of Tongji Hospital (TJH‐202110039).

## Conflict of Interest

The authors declare no conflict of interest.

## Author Contributions

X.L. and M.H. contributed equally to this work. B.Z., Z.L., and X.C. conceived this study; B.Z. and Z.L. provided financial and administrative support; X.L. and M.H. conducted most experiments; H.Z. and H.L. collected the public data and patients’ data of Tongji cohort; X.L. wrote the manuscript. All authors have read and approved the final manuscript.

## Supporting information



Supporting Information

## Data Availability

All data generated or analyzed are included in the article and its supplementary files, and available from the corresponding author upon request.
