# Supplementary material for: m6A‐Mediated TMCO3 Promotes Hepatocellular Carcinoma Progression by Facilitating the Membrane Translocation and Activation of AKT
Source: Adv Sci (Weinh). 2025 Apr 26;12(27):2504187. doi: 10.1002/advs.202504187 (PMC12279241; doi:10.1002/advs.202504187)
Supplement: Supplementary file 1 — Supporting Information [file ADVS-12-2504187-s001.docx]

**Supplementary Material**

**Supplementary Methods and Materials**

**Sample collection**

Human HCC tumor tissues and adjacent-normal tissues were collected from HCC patients between 2014 to 2016 at the Hepatic Surgery Center, Tongji Hospital of Huazhong University of Science and Technology (HUST). The tissue microarray of 123 pairs of HCC tissues with their prognosis data were collected from Hepatic Surgery Center, Tongji Hospital of Huazhong University of Science and Technology. Ethical approvals were acquired from the Ethics Committee of Tongji Hospital according to the Declaration of Helsinki Principles. Each patient’s written consent was obtained before the collection of specimens.

**Cell Lines and Cell Culture**

The THLE-3, Hep3B, HLF, HepG2 and Alex were purchased from China Center for Type Culture Collection (CCTCC, Wuhan, China). HCC cells lines MHCC97H and LM3 were obtained from Liver Cancer Institute, Zhongshan Hospital, Fudan University (Shanghai, China). These cell lines were maintained as monolayer cells in Dulbecco’s modified Eagle’s medium (DMEM) (HyClone) with 10% fetal bovine serum (FBS) (Gibco, Grand Island, NY) at 37 °C in 5% CO2.

AuGCT Biotech (Wuhan, China) designed and purchased anti-sense oligonucleotides (Asos) to knock down the TMCO3(mouse) gene and other m^6^A regulators, as well as negative control Asos. Lipofectamine 3000 Reagent was used to transfect Asos according to the manufacturer’s instructions (Invitrogen, USA). The full-length coding sequences of TMCO3 was provided by Tsingke Biological Technology (Beijing, China) were cloned into the pcDNA3.1 vector (Invitrogen, USA).

**m^6^A MeRIP-Seq**

The m^6^A MeRIP-Seq service was provided by CloudSeg Inc. (Shanghai, China). Total RNA was subjected to immunoprecipitation with the GenSeq® m^6^A MeRIP Kit (GenSeg, Inc.) by following the manufacturer's instructions. Briefly, RNA was randomly fragmented to ~200 nt by RNA Fragmentation Reagents. Protein A/G beads were coupled to the m6A antibody by rotating at room temperature for 1 h. The RNA fragments were incubated with the bead-linked antibodies and rotated at 4°C for 4 h. After incubation, the RNA/antibody complexes were washed for several times, and then, captured RNA was eluted from the complexes and purified. RNA libraries for IP and input samples were then constructed with GenSeg® Low Input Whole RNA Library Prep Kit (GenSeg, Inc.) by following the manufacturer's instructions. Libraries were qualified using Agilent 2100 bioanalyzer (Agilent) and then sequenced.

**Cell Proliferation Assay**

HCC cell proliferation was determined using the CCK-8 Cell Counting Kit (Vazyme, China, A311–01). After transfection, MHCC-97H, HLF cells were seeded into 96-well plates (1000 cells/well). Then, according to the manufacturer’s protocol, the cells Culture the cells and record the 450nm absorbance at 24, 48, 72, 96, and 120hours respectively. All experiments were performed 3 times, and the results are shown as the average value of + SD.

**Colony formation assay**

Add MHCC-97H, HLF cells to each well of a 6-well plate (1500 cells/plate), and continue to culture for 14 days in DMEM containing 10% FBS. Change the medium every 7days. After two weeks, the cells were washed twice with PBS, fixed with 4% formaldehyde for 15minutes, and stained with crystal violet for 15minutes. Count and analyze cell clones.

**Transwell cell migration and invasion assays**

24-well Transwell plates and trans-well chambers (pore size, 8 μm; Corning, NY, USA) were used in cell migration and invasion assays. Additionally, chambers were coated with 50 μl 1:3 mixture of Matrigel (BD Biosciences, NJ, USA) and non-FBS DMEM for 1 hour at 37˚C to perform invasion assays. Cells were harvested and seeded into the upper chambers while DMEM containing 10% FBS was added into the lower chambers. After incubating for 24h, the chambers were fixed and stained at room temperature. Numbers of trans-well cells were counted under an optical microscope. All experiments were repeated for three times.

**Wound healing assay**

6-well Transwell plates were used in wound healing assay. Cells were cultured in a non-FBS DMEM for starvation for 24 hr. The monolayer was scratched by a pipette tip. The cells were allowed to migrate at the indicated time, and test the distance between the scratch edges.

**RT–qPCR**

Total RNA was extracted from cells using FastPure Cell Total RNA Isolation Kit V2 (RC112-01, Vazyme, Nanjing, China). Total RNA was converted into cDNA using the HiScript III RT SuperMix (R323-01, Vazyme, Nanjing, China). RT-qPCR analysis was performed with ChamQ SYBR qPCR Master Mix (Q711-02, Vazyme, Nanjing, China) according to the manufacturers' protocols, GAPDH was used as the endogenous control. Relative analysis was performed using the comparative CT (2-ΔΔCT) method. Each assay was independently repeated three times.

**Western blot and antibodies**

It was separated for 2 hours on a 10% SDS-PAGE gel (20325ES62, YEASEN, Shanghai, China) before being transferred to a PVDF membrane (0.45μm, Roche). The membrane was blocked for 1hour at room temperature with 5% skim milk in TBST, then the primary antibody was added and incubated for overnight at 4°C. The membrane was then rinsed three times with TBST before being incubated for 1 h at 37°C with HRP-conjugated goat anti-rabbit or goat anti-mouse immunoglobulin G secondary antibody (Serviecbio, Wuhan, China). TBST washed the membrane three times more. The target western blot was detected using the ECL method (Bio-Rad, USA).

Abcam provided the TMCO3 (ab154581) and Thiophosphate ester (ab92570) antibodies. Atlas provided the TMCO3 (HPA048126) antibody. Proteintech provided the METTL3 (15073-1-AP), METTL14 (26158-1-AP), WTAP (10200-1-AP), FTO (27226-1-AP), GAPDH (60004-1-Ig), IGF2BP2 (11601-1-AP). Cell Signaling Technology provided the AKT (#4691), Phospho-Akt (Ser473) (#4060), Phospho-Akt (Thr308) (#13038), Phospho-(Ser/Thr) Phe (#9631), c-MET (#8198) β-Catenin (#8480), GST-Tag (#2625) antibodies. ABclonal provided the Na+/K+-ATPase (A11683), IGF2BP1 (A22246), IGF2BP3 (A23295), YTHDF1 (A23773), YTHDC2 (A24219), EIF3A (A0573) antibody.

**Anti-Phospho-TMCO3-S85 production**

Peptide synthesis and anti-Phospho-TMCO3-S85 antibody preparation were performed by ABclonal (Wuhan, China). Briefly, a peptide KDVGLS(p)DEEKL-C was synthesized, and polyclonal antibodies against the Phospho-TMCO3-S85 peptide were obtained from four inoculated rabbits. Antibodies were purified using affinity chromatography on columns containing the corresponding peptides. According to our tests, final purified antibody (E28609) was the correct antibody to recognize the Phospho-TMCO3-S85 peptide. The WB dilution: 1/500-1/1000.

**Co-Immunoprecipitation**

The specific reagent components were seen in our previous research. Briefly, cells were collected and lysed in IP-lysis buffer (MB9900, Meilunbio, Dalian, China). Supernatants were collected by centrifugation (15,000 g, 15 min, 4°C), and the cleared supernatants were incubated with the indicated antibodies (1 µg/ml) for overnight at 4°C. The complex was added with protein A/G magnetic beads (L-1004, Biolinkedin, Shanghai, China) and incubated for 3 hours at 4°. The complex was washed 5-7 times with IP-wash buffer and detected through WB.

**PIP_3_-pull down assay**

For PIP_3_-pull down assay, FLAG-TMCO3 and HA-AKT proteins and 20 μL PIP_3_-coated beads (Echelon Biosciences) were mixed in 1 ml binding buffer (50 mM Tris, 150 mM NaCl, 0.05% NP-40, pH 7.5) and rotated for 15 min at room temperature. After centrifugation at 13,000 rpm for 10 min, pellets were resuspended in 1 ml binding buffer and washed 3 times. Pellets and flowthrough were analyzed by SDS-PAGE and immunoblotted with HA antibody.

**Protein–lipid binding assay**

Proteins were spotted on Membrane Lipid Strips (Echelon Biosciences) according to the manufacturer’s instructions. To block non-specific binding, lipid strips were pre-incubated with binding assay buffer (3% fatty acid-free BSA in PBS) for 1h at room temperature. Then the strips were incubated with protein (2 μgml^−1^) diluted in binding assay buffer for 1h at room temperature and then washed three times (6min each time) with wash buffer (0.1% Tween-20 in PBS). Membrane-bound proteins were detected by probing the lipid strips with corresponding primary antibodies diluted in binding assay buffer for 1h at room temperature, followed by incubation for 1h with horseradish-peroxidase-conjugated secondary antibody diluted 1:2000 in binding assay

buffer. After washing three times with wash buffer, proteins were visualized using ECL kit.

**Purification of FLAG-TMCO3 and FLAG-TMCO3-S85A proteins from 293 T cells**

Flag-TMCO3 and FLAG-TMCO3-S85A proteins were purified from 293T cells. The anti-Flag Magnetic Beads (HY-K0207-1) and the 3xFlag peptide (HY-P0319) were purchased from MedChemExpress. The details are described in the product manual.

**Purification of GST-AKT protein from bacteria**

Recombinant GST-conjugated AKT/AKT^T308A^/AKT^S473A^ domain was generated by transforming the BL21 (DE3) *E.coli* strain with pGEX-AKT, respectively. The cultured bacteria were grown at 37 °C to an O.D. 0.8, and then the protein expression was induced for 12–16 h by adding 0.1 mM IPTG at 16 °C with vigorous shaking. Recombinant proteins were purified from harvested pellets and re-suspended in 10 ml EBC buffer for sonication. Insoluble proteins and cell debris were discarded, and the supernatant was incubated with 50 µl 50% Glutathione-sepharose slurry for 3 h at 4 °C. The Glutathione beads were washed 3 times with PBS buffer and stored at 4 °C in PBS buffer containing 10% glycerol or eluted by elution buffer.

**GST-pull down assay**

The Ripa buffer washed Glutathione Sepharose 3-5 times, and centrifuged at 4000rpm at 4℃ for 1min after each suspension. The washed magnetic beads were added to the 480 µl GST fusion protein supernatant and incubated at rotator at 4℃ for 1h and 4000rpm at 4℃ for 1min. After washing with 800 µl RIPA lysate for 3-5 times (5min at 4℃ at rotator), supernatant containing purified Flag-tagged proteins (From 293T, 10cm dish) was added into the GST negative control and GST fusion protein beads respectively. Incubate at 4℃ for overnight. Wash the magnetic beads three times like before. Added 20-50 µl 1xLoading Buffer, boiled at 95℃ for 10min, centrifuged at 12000rpm for 1min, supernatant was used for WB analysis.

**Kinase activity assay**

The concentrations of remaining ATP in reactions were measured using Kinase-Lumi Plus Luminescent Kinase Assay Kit (Beyotime Biotechnology). The kinase, ATP, substrate and kinase buffer were mixed with a total volume of 50 µl, and 50 µl of reaction reagent was added into the mixture to incubate for 10 min at 25°C. Then, chemiluminescent was detected by luminometer. The enzymatic activity was calculated on the basis of ATP consumption.

***In vitro* kinase assay**

Protease inhibitors and phosphatase inhibitors are pre-added before the use of the kinase reaction buffer to prevent protein degradation and loss of phosphorylation modification. 10 µg GST-AKT/AKT^T308A^/AKT^S473A^ purified protein and 0.5 µg purified FLAG-TMCO3/FLAG-TMCO-S85A proteins were added to 50 µL kinase reaction buffer, then 50 µM ATP-γ-S was added, and incubated at 30℃ for 30min-60min. Then, 2.5 mM of p-Nitrobenzyl mesylate (PNBM) was added to the sample and incubated at room temperature for 1h. After the reaction was completed, loading buffer was added to denaturate the sample at 95℃ for 10min, and WB analysis was performed subsequently. Phosphorylation was determined by Anti-Thiophosphate ester antibody.

**Immunofluorescence**

Cells were fixed with 4% paraformaldehyde for 15 min, followed by permeabilization with 0.5% Triton X-100 for 20 min at room temperature. Primary antibodies (0.2 µg/ml) were added for 2 hr at room temperature post blocking with 5% bovine serum albumin for 1 hr. FITC or DyLight549-conjugated Goat anti-Mouse IgG and DyLight549 or DyLight649-conjugated Goat anti-Rabbit IgG were used as secondary antibodies. Nuclei were counterstained with DAPI. F-actin stress fibers were stained with Alexa Fluor 555-conjugated Phalloidin (Life Technologies). Images were taken by confocal laser-scanning microscopy on a Nikon Digital ECLIPSE C1 system (Nikon Corporation).

**Immunohistochemistry**

Immunohistochemical staining for tissues was performed by using the polymer HRP detection system (Zhongshan Goldenbridge Biotechnology). The paraffin sections were deparaffinized, antigen retrieval, blocked endogenous peroxidase, then blocked with 5% bovine serum albumin, incubated with primary antibody and secondary antibody, and finally detected by DAB, the detailed steps were carried out as described previously. All ISH or IHC samples were scored by two independent pathologists. The IHC score was divided into two parts, the staining intensity score and staining positive area score. The staining intensity was scored as follows: 0: negative, 1 point: weak positive (light brown), 2 points: medium positive (brown), and 3 points: strong positive (dark brown). The positive area of staining was scored as follows: 0:0%, 1:10-25%, 2:26-50%, 3:51-75%, and 4:76-100%. We divided them into high expression group according to the score greater than 6, and low expression group according to the score less than or equal to 6.

**Multiplex immunohistochemistry**

The TSA Fluorescence Triple Staining Kit (RK05903) was purchased from ABclonal. The steps are the same as IHC. After the second antibody is washed, the corresponding color of fluorescent dye is prepared and incubated at room temperature for 15-20 minutes. Wash 3 times with PBS at room temperature. If any other indicators needed to dye, go back to the beginning of the antigen repair step. After all targets were dyed, DAPI was dyed for 10 minutes, washed 3 times with PBS at room temperature, sealed and stored at 4°.

**Cytoplasm and membrane protein fractionation assay**

Mem-PER™ Plus membrane protein extraction kit (89842) was provided by ThermoFisher, and the procedure is as follows. Resuspend 5 × 10^6^ cells in the growth media by scraping the cells off the surface of the plate with a cell scraper. Centrifuge harvested cell suspension at 300 × g for 5 minutes, and wash cell pellet with 3mL of Cell Wash Solution and centrifuge at 300 × g for 5 minutes. Then, carefully remove and discard the supernatant. Resuspend the cells in 1.5mL of Cell Wash Solution and transfer to a 2mL centrifuge tube. Centrifuge at 300 × g for 5 minutes and discard supernatant. After that, add 0.75mL of Permeabilization Buffer to the cell pellet. Vortex briefly to obtain a homogeneous cell suspension. Incubate 10 minutes at 4°C with constant mixing. Next, centrifuging permeabilized cells for 15 minutes at 16,000 × g. Carefully remove the supernatant containing cytosolic proteins and transfer to a new tube. Add 0.5mL of Solubilization Buffer to the pellet and resuspend by pipetting up and down. Incubate tubes at 4°C for 30 minutes with constant mixing. Centrifuge tubes at 16,000 × g for 15 minutes at 4°C. Transferring supernatant containing solubilized membrane and membrane-associated proteins to a new tube. Proceed to downstream application. Immediately use cytosolic and membrane fractions stored on ice or store aliquots at 80°C for future use.

**RNA immunoprecipitation (RIP)**

RIP assay was performed to using Magna RIP™ RNA-Binding Protein Immunoprecipitation Kit (Millipore, Germany). qRT–PCR was subsequently performed to analyze the enriched RNA.

**MeRIP‑qPCR assay**

The m^6^A immunoprecipitation (MeRIP) procedure was performed according to instructions issued by the manufacturer using a Magna MeRIP™ m^6^A kit (#17–10499, Merck Millipore, MA). Briefly, purified mRNA was digested by DNase I and then fragmented into ∼100nt using RNA fragmentation reagent and incubated at 94 °C. After fragmenting, the stop buffer was added, following which standard ethanol precipitation was performed and collected. The anti-m6 A antibody for 12μg was pre-incubated with 50μl beads in IP buffer (150mM NaCl, 0.1% NP-40, 10mM Tris–HCl, pH7.4) at room temperature for 1h. Next, 6μg of fragment mRNAs were added to the antibody-beads mixture and incubated at 4°C for 4h on a rotator. After adequate washing, immunoprecipitated mixture was digested using high concentration of proteinase K, and the bound RNAs were extracted using phenol-chloroform method and ethanol precipitation and were used for qPCR analysis.

**RNA stability assay**

HCC cells were treated as follows: 6-well plates were used to seed the cells overnight, and actinomycin D (5μg/mL, HY-17559, MedChemExpress) was used to treat them for 0, 2, 8h. Total RNA was isolated using TRIzol and quantified by qRT-PCR. Group expression of the mRNA at the indicated times was calculated and normalized by GAPDH.

**Dual Luciferase Report Assays**

The representative length of TMCO3 gene, the mutant of the TMCO3 were cloned into the psiCHECKTM-2-vector (Promega, Madison, WI, USA) at a site immediately downstream of the Renilla luciferase gene. Cells were seeded at a density of over 50% into 24-well plates for 24 h and co-transfected with relative reagents. 48h after transfection, the luciferase activity was measured using the Dual Luciferase Reporter Assay Kit (RL101-01, Vazyme, Nanjing, China). Experiments were repeated three times.

**AAV8 treatment**

AAV8 containing scramble or shTMCO3 sequences under the control of TBG promoter was produced by DesignGene Biotechnology (shanghai，China). For prophylactic treatment, a 50 µl dose of AAV8 (10^13^ genome copies/mouse) was delivered by tail vein.

**Animal experiments and treatments**

For the combined treatment of the HTVi induced HCC, AAV-shTMCO3 (mouse) reagent was injected into the tail vein of the corresponding experimental group of mice for one time in the third week after the injection of plasmid in the tail vein. The MK2206 (S1078) inhibitors were provided by Selleck. In the MK2206 treatment group and the combination treatment group, MK2206 (40mg/kg) were administered intragastrically every three days from 4^th^ to 6^th^ week before the mice were sacrificed. For the combined treatment of the orthotopic xenograft models in nude mice, AAV-shTMCO3 (human) reagent was injected into the tail vein of the corresponding experimental group of mice for one time in the third week after the cells were injected in liver. In the MK2206 treatment group and the combination treatment group, MK2206 (100mg/kg) were administered intragastrically every two days from 3^th^ to 5^th^ week before the mice were sacrificed.

**Supplementary figures**

**Supplementary Figure 1**

**
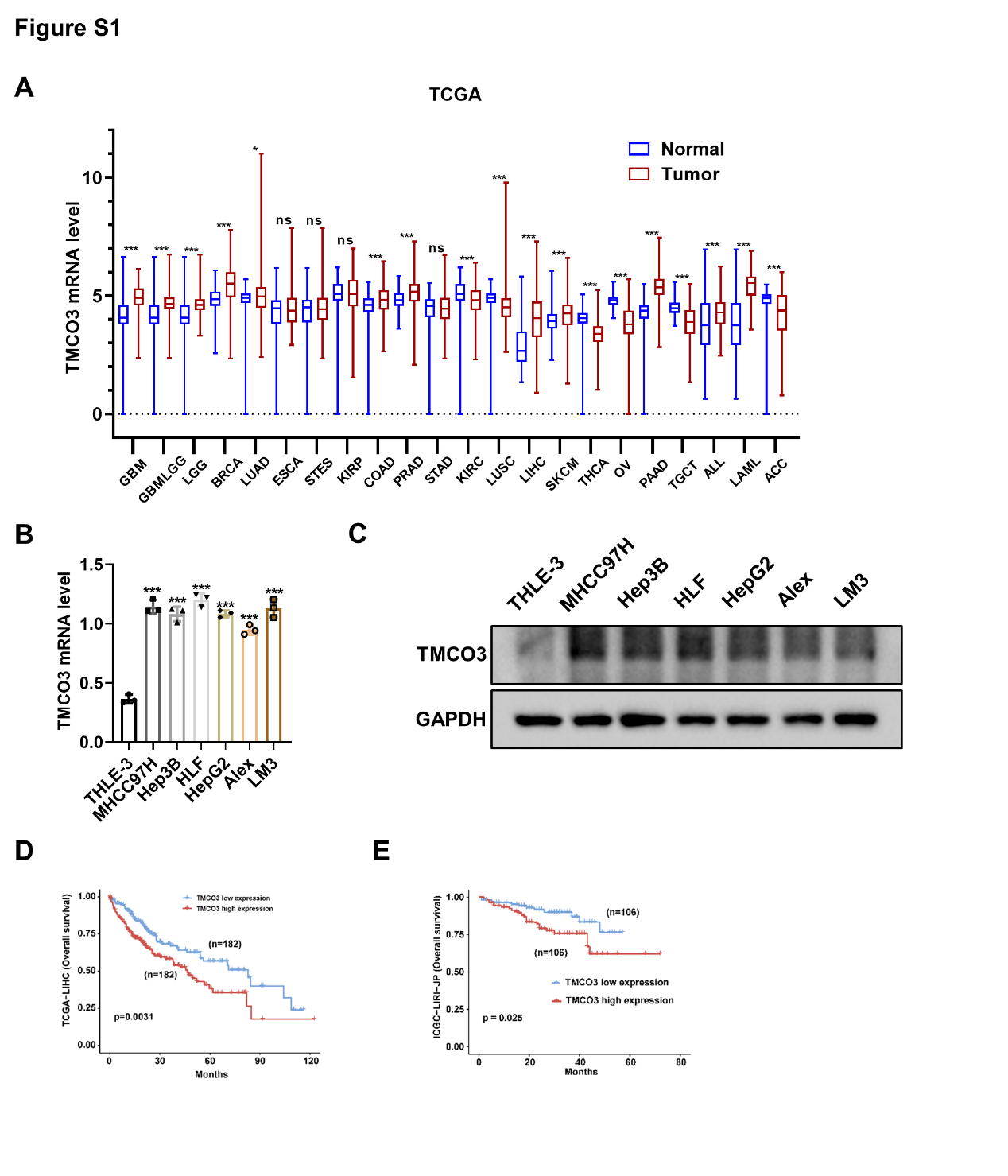
**

**Fig S1. TMCO3 presents a high m^6^A modification level and high expression level in HCC.**

(**A**) The statistical chart showed that TMCO3 mRNA levels in normal and tumor tissues of pan-cancer in the TCGA database. (**B**) The mRNA levels of TMCO3 in normal liver cell and other HCC cell lines. (**C**) The protein levels of TMCO3 in normal liver cell and other HCC cell lines. (**D**) The overall survival with TMCO3 low expression or TMCO3 high expression in TCGA-LIHC database. (**E**) The overall survival with TMCO3 low expression or TMCO3 high expression in ICGC-LIRI-JP database.

**Supplementary Figure 2**

**
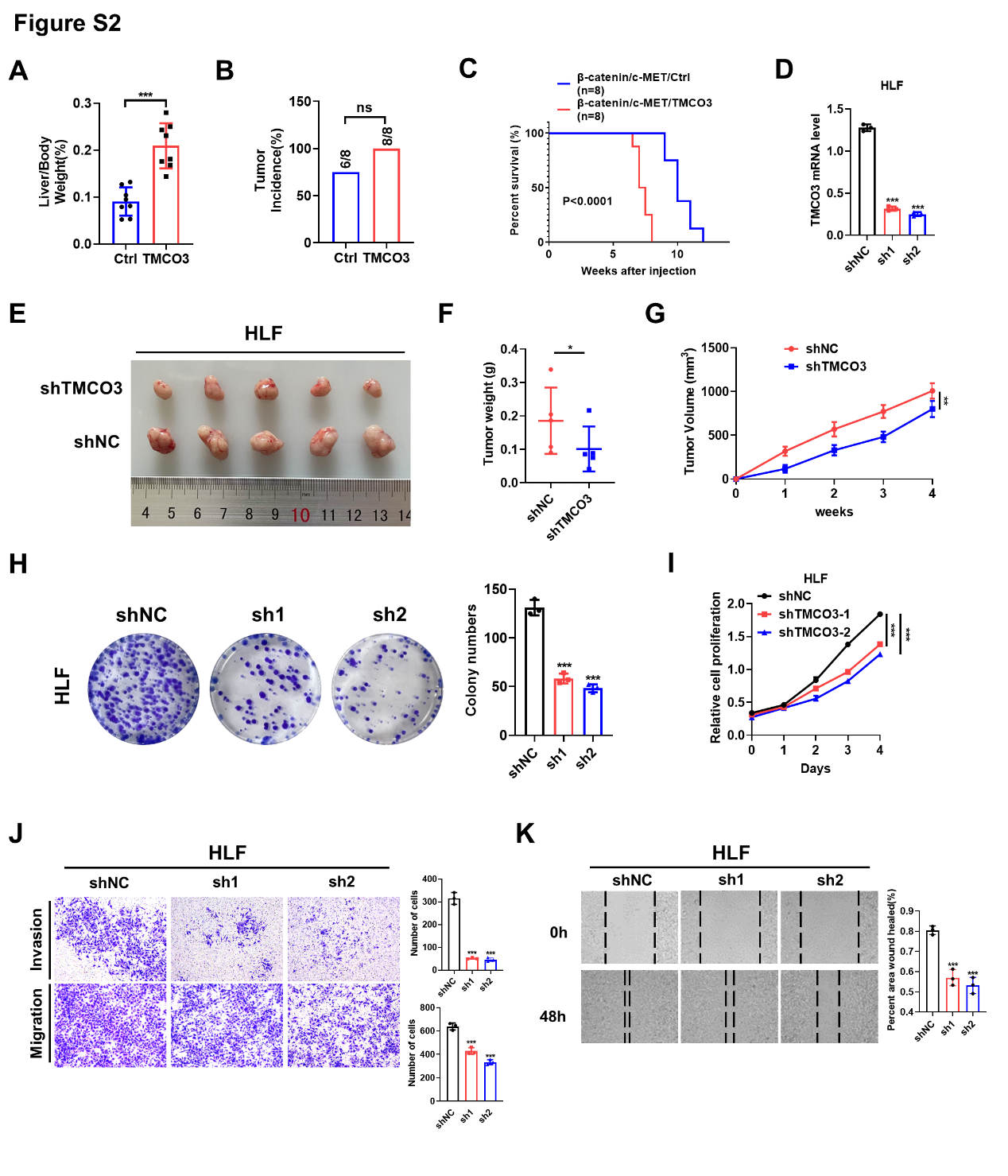
**

**Fig S2. TMCO3 promotes HCC proliferation and metastasis in vivo and in vitro.**

(**A**) The statistics chart of liver/body weight ratio in Ctrl group and TMCO3 group. (**B**) The tumor incidence in Ctrl group and TMCO3 group. (**C**) The survival rate of mice in β-catenin+c-MET+PT3-Ctrl group and β-catenin+c-MET+PT3-TMCO3 (Mouse) group. (**D**) The mRNA levels of TMCO3 in HLF cells after knockdown of TMCO3. (**E**) The gross image of subcutaneous tumors in HLF-Ctrl and HLF-shTMCO3 group. (**F**) The tumor weights of subcutaneous tumors in HLF-Ctrl and HLF-shTMCO3 group. (**G**) The tumor volumes of subcutaneous tumors in HLF-Ctrl and HLF-shTMCO3 group. (**H**) Colony formation assays of HLF cells in shNC, sh1 and sh2 groups. (**I**) CCK-8 assays of HLF cells in shNC, sh1 and sh2 groups. (**J**) Migration and invasion assays of HLF cells in shNC, sh1 and sh2 groups. (**K**) Wound healing assays of HLF cells in shNC, sh1 and sh2 groups.

**Supplementary Figure 3**

**
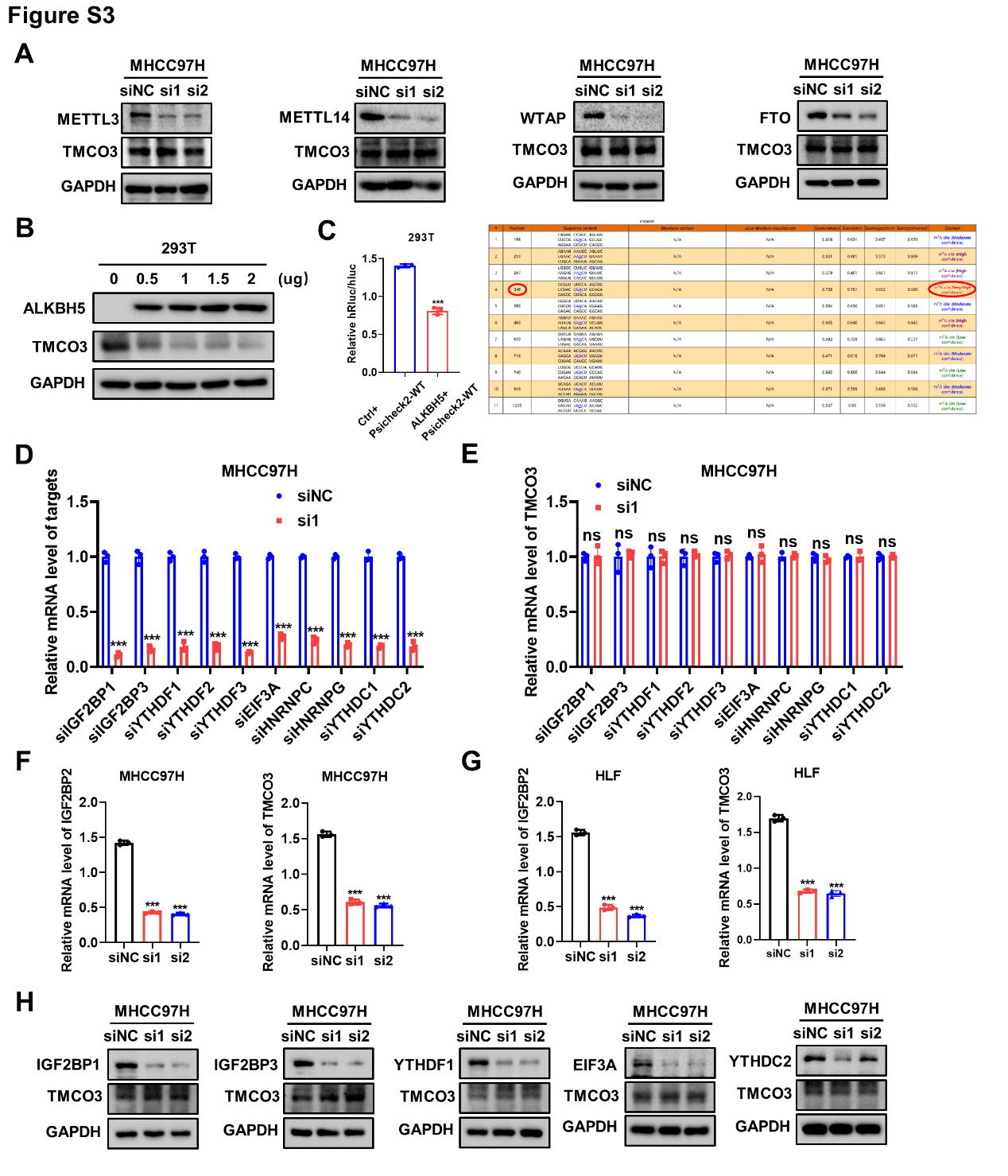
**

**Fig S3. ALKBH5 downregulates TMCO3 in an m^6^A-dependent manner.**

(**A**) The western blot results showed the protein levels of TMCO3 after knockdown of METTL3, METTL14, WTAP and FTO. (**B**) The western blot results showed the protein levels of TMCO3 after transfection of ALKBH5 by way of increased concentration. (**C**) The relative luciferase activities of Ctrl group and ALKBH5 group and the schematic diagram of m^6^A potential sites of TMCO3 on the SRAMP analysis. (**D, E**) The mRNA levels of targets in MHCC97H cells after knockdown of IGF2BP1, IGF2BP3, YTHDF1, YTHDF2, YTHDF3, EIF3A, HNRNPC, HNRNPG, YTHDC1 and YTHDC2. (**F, G**) The mRNA levels of IGF2BP2 and TMCO3 in MHCC97H and HLF cells after knockdown of IGF2BP2. (**H**) The protein levels of TMCO3 in MHCC97H cells after knockdown of IGF2BP1, IGF2BP3, YTHDF1, EIF3A, YTHDC2.

**Supplementary Figure 4-1**

**
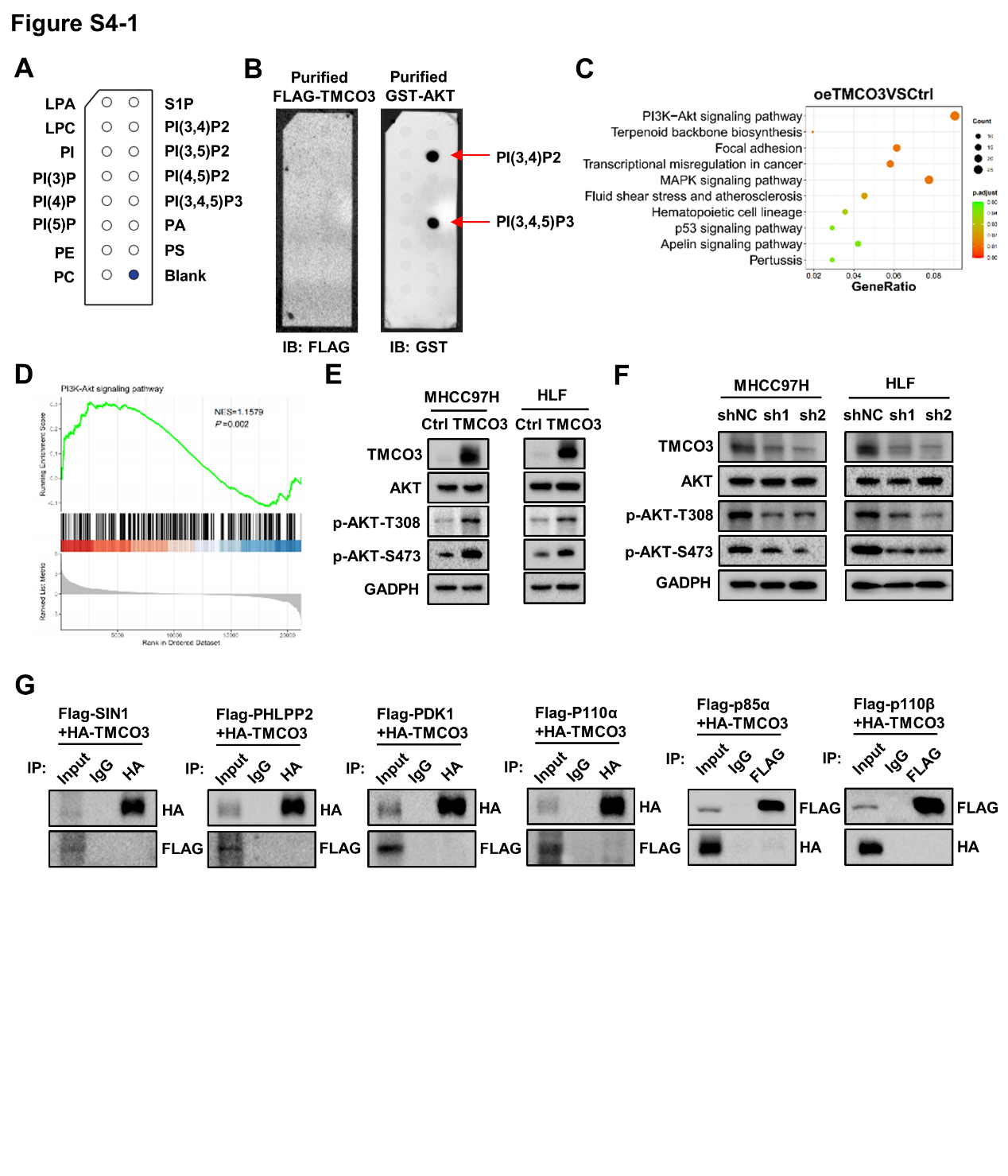
**

**Fig S4-1. TMCO3 binding with AKT directly and increases AKT phosphorylation in a PI3K dependent manner.**

(**A, B**) Membranes displaying lipids were incubated with indicated proteins and binding was assessed by blotting for FLAG antibody and GST antibody. (**C**) The drawing of KEGG pathway enrichment analysis in TMCO3-overexpression MHCC97H cells compared with control MHCC97H cells. (**D**) The drawing of GSEA in TMCO3-overexpression MHCC97H cells compared with control MHCC97H cells. (**E**) The western blots results showed the protein levels of relative targets of MHCC97H and HLF cells in Ctrl group and TMCO3 group. (**F**) The western blots results showed the protein levels of relative targets of MHCC97H and HLF cells after knockdown of TMCO3. (**G**) CO-IP assays showed the negative binding relationship between those proteins.

**Supplementary Figure 4-2**


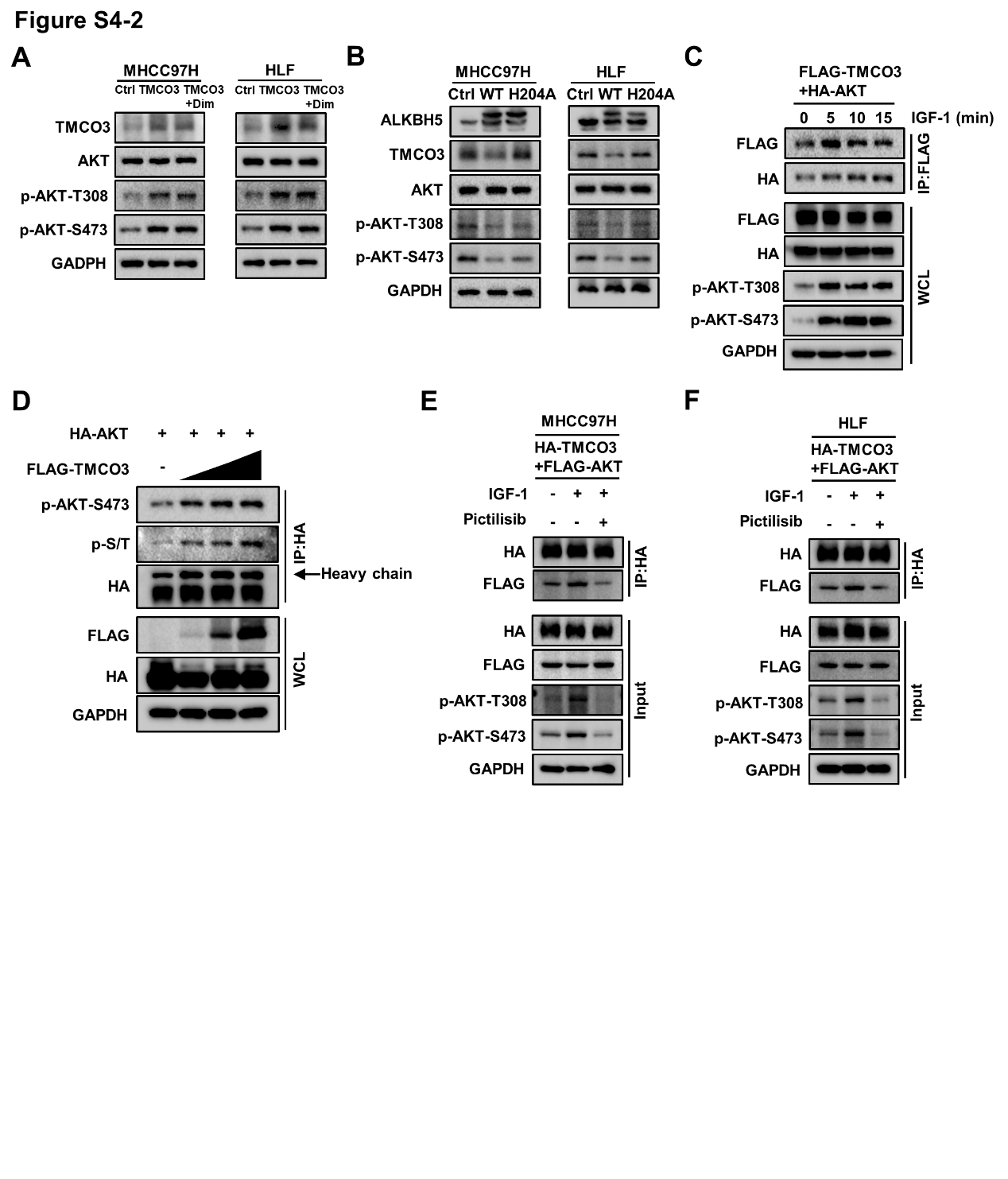


**Fig S4-2. TMCO3 binding with AKT directly and increases AKT phosphorylation in a PI3K dependent manner.**

(**A**) The western blots results showed the protein levels of TMCO3 and relative targets between these three groups in MHCC97H and HLF cells. (**B**) The western blots results showed the protein levels of relative targets between these three groups in MHCC97H and HLF cells. (**C**) IP assays showed the interaction between TMCO3 and AKT after IGF-1 stimulation in time gradient. (**D**) IP assays showed the interaction between TMCO3 and AKT after transfection of TMCO3 in concentration gradient. (**E, F**) IP assays showed the interaction between TMCO3 and AKT in MHCC97H and HLF cells in Ctrl, IGF-1, IGF1+Pictilisib groups.

**Supplementary Figure 5**

**
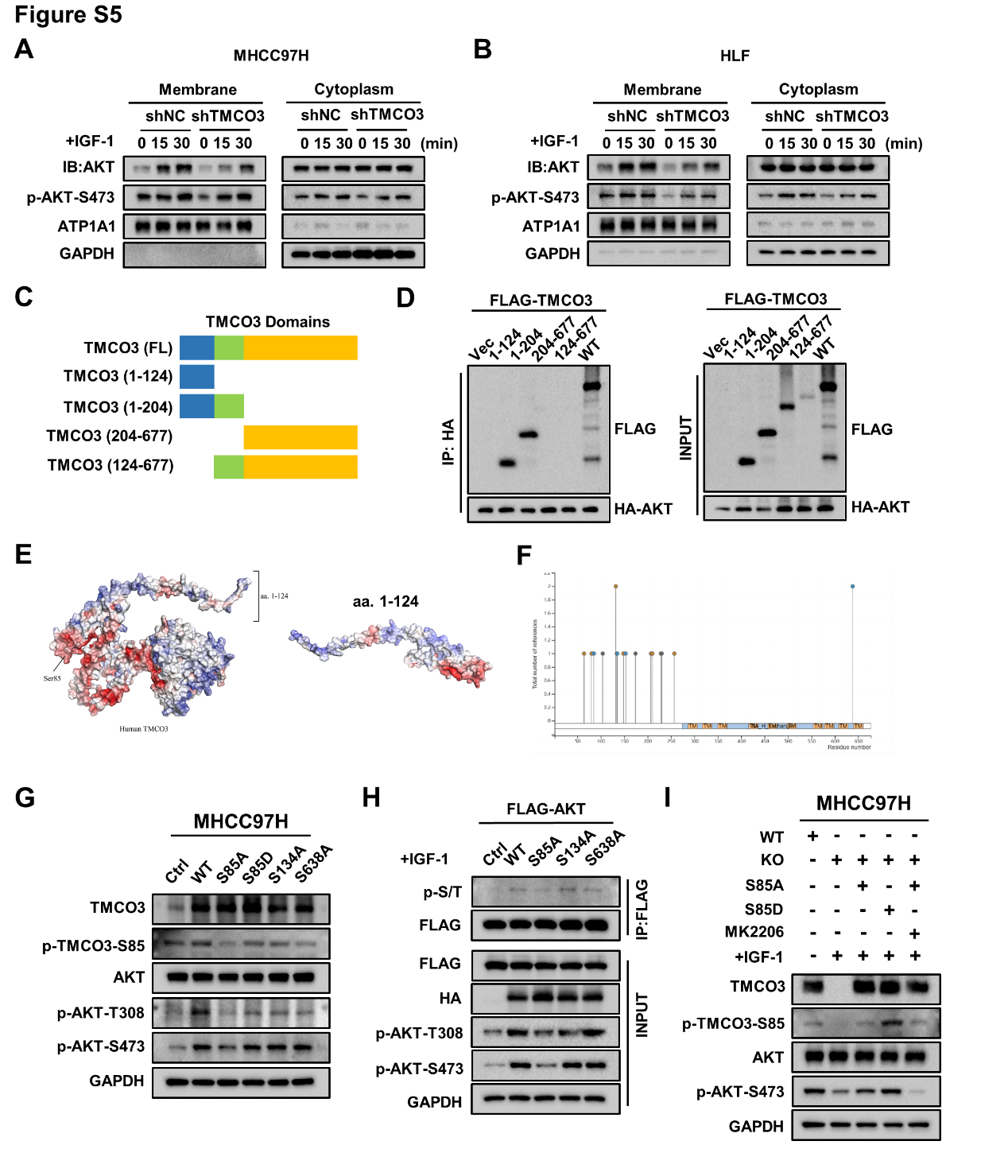
**

**Fig S5. The IGF-1-stimulated phosphorylation on the serine 85 site of TMCO3 facilitates the membrane localization and activation of AKT.**

(**A**) The Cytoplasm and membrane protein fractionation assay was performed in MHCC97H cells after knockdown of TMCO3 or control. (**B**) The Cytoplasm and membrane protein fractionation assay was performed in HLF cells after knockdown of TMCO3 or control. (**C**) Schematic diagram of the truncated region of TMCO3. (**D**) IP assays showed the binding of different truncated regions of TMCO3 to AKT. (**E**) Schematic diagram of the 3D structure of TMCO3 and the amino acids 1-124. (**F**) The schematic diagram of the predicting phosphorylation sites from the Cell Signaling Technology (CST) MS database. (**G**) The western blot results showed the protein levels of targets after transfection of TMCO3-WT, S85A, S85D, S134A, S638A. (**H**) IP assays showed the P-S/T level after co-transfection of FLAG-AKT and other plasmids. (**I**) The western blot results showed the protein levels of targets in relative groups.

**Supplementary Figure 6**

**
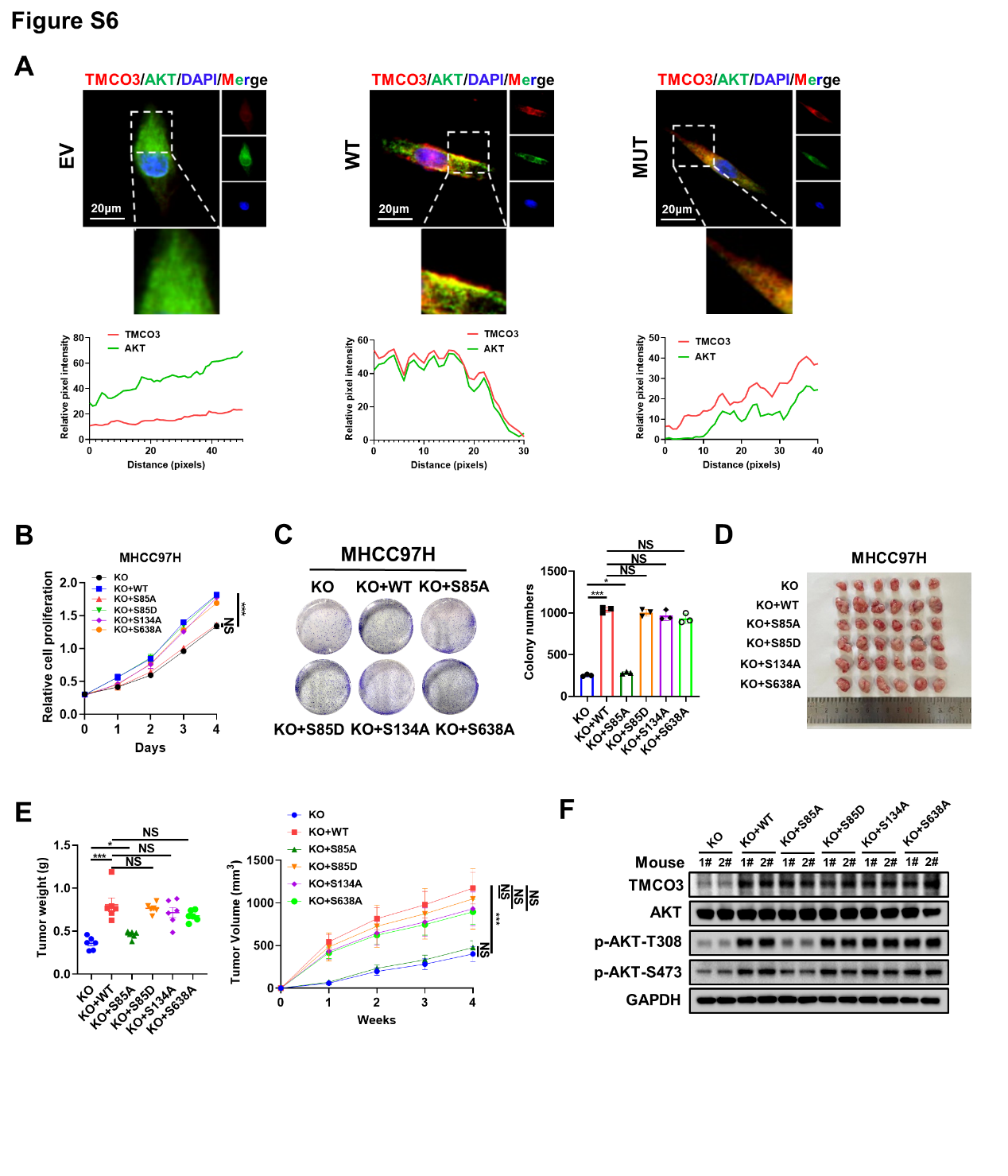
**

**Fig S6. Mutation on the Serine 85 of TMCO3 inhibits AKT membrane location and kinase activity, ultimately impeding HCC progression.**

(**A**) The Cytoplasm and membrane protein fractionation assay was performed in HLF cells after overexpression of TMCO3-WT, TMCO3-MUT (S85A) or control. (**B**) CCK-8 assays of MHCC97H cells in relative groups. (**C**) Colony formation assays of MHCC97H cells in relative groups. (**D**) The gross image of subcutaneous tumors in relative groups. (**E**) The tumor weights and tumor volumes of subcutaneous tumors in relative groups. (**F**) The western blot results showed the protein levels of relative targets of subcutaneous tumors in relative groups.

**Supplementary Figure 7-1**

**
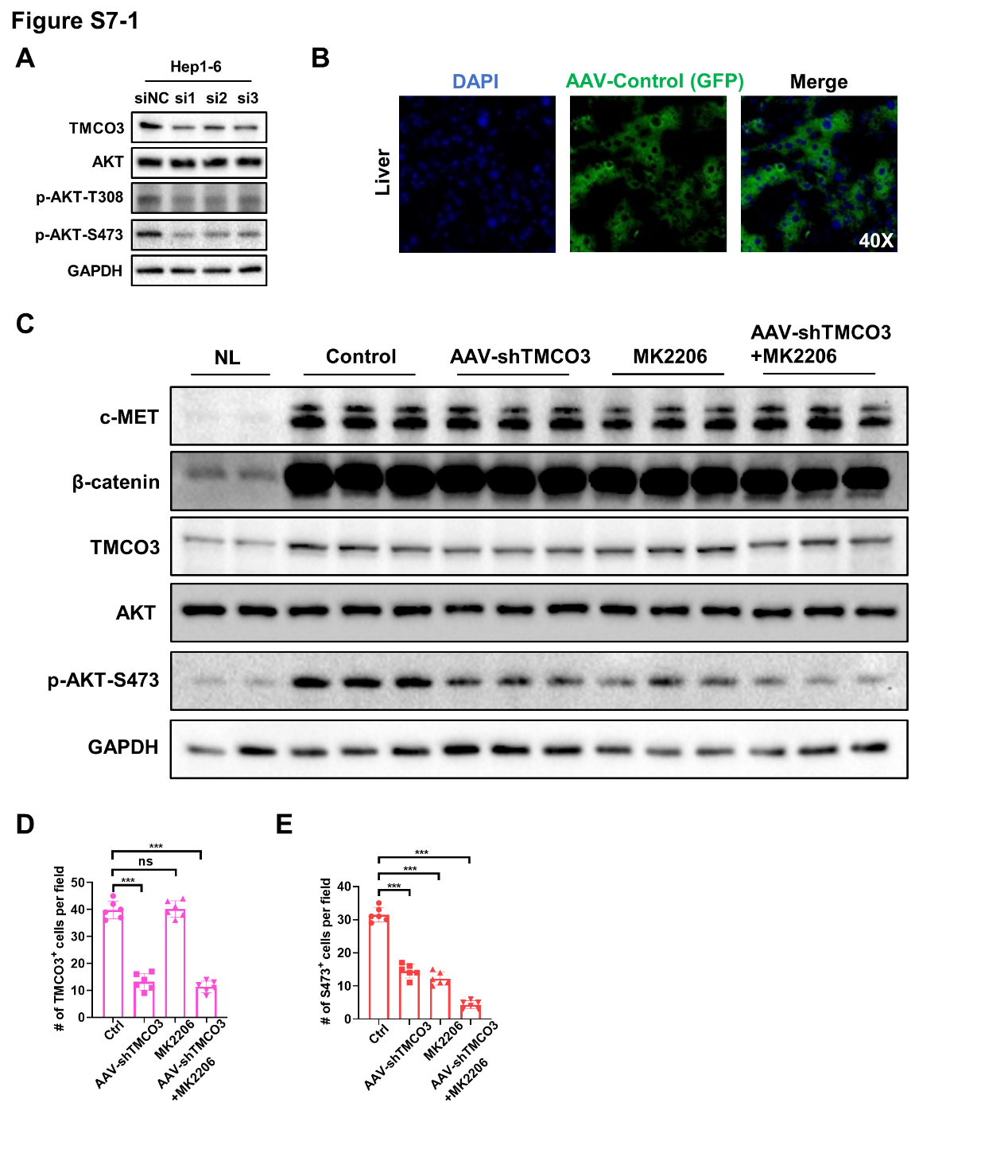
**

**Fig S7-1. Combined treatment with AAV-shTMCO3 and MK2206 dramatically decreases AKT phosphorylation and inhibits tumorigenesis in mouse model.**

(**A**) The western blot results showed the protein levels of TMCO3 and other targets after knockdown of TMCO3 (mouse) in Hep1-6 cells. (**B**) Immunofluorescence of freezing microtome showed that AAV-Control could target the liver. (**C**) The western blot results verified the expression levels of hydrodynamic plasmids and other targets in mice. (**D**) The number of TMCO3 stained cells in each treatment group. (**E**) The number of P-AKT-S473 stained cells in each treatment group.

**Supplementary Figure 7-2**

**
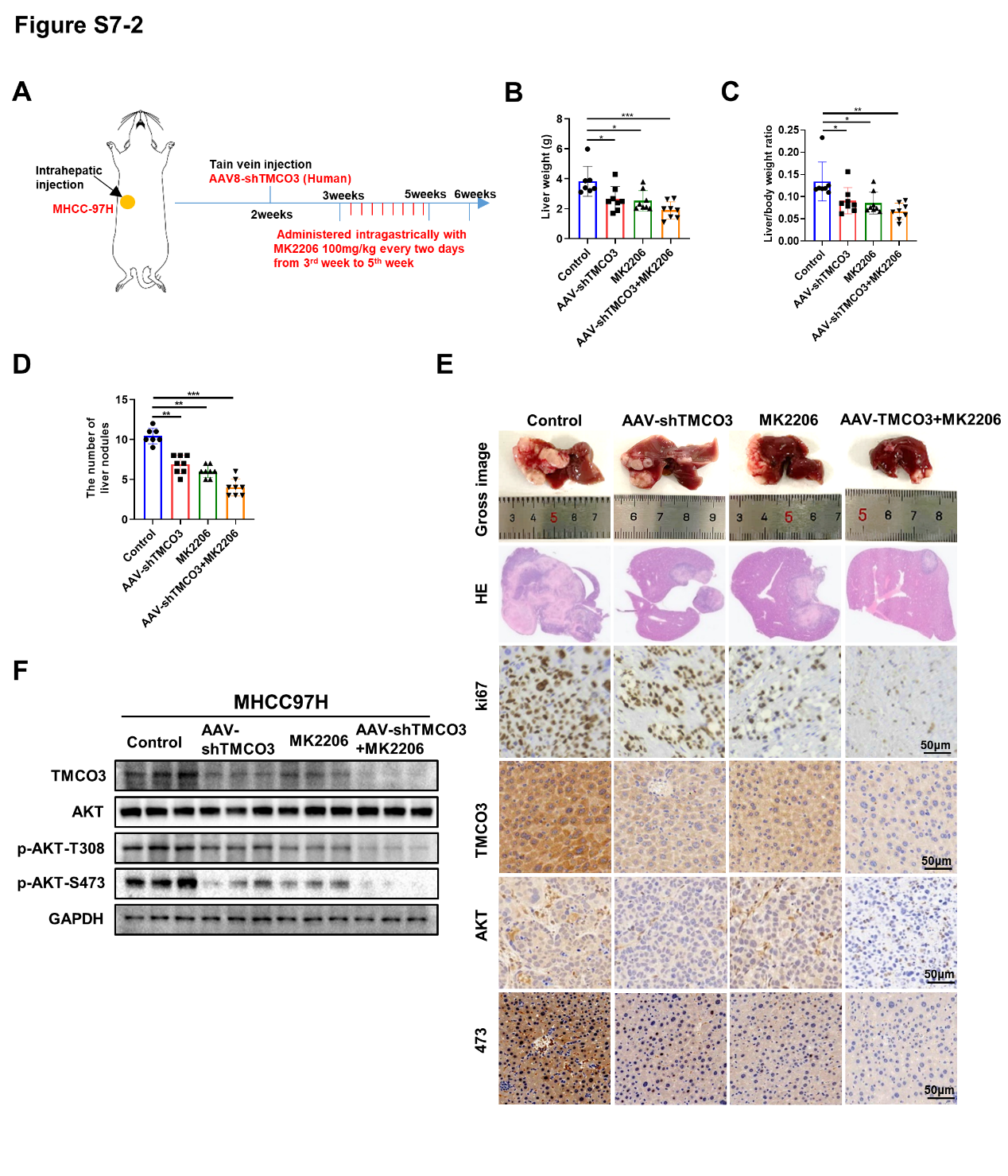
**

**Fig S7-2. Combined treatment with AAV-shTMCO3 and MK2206 dramatically decreases AKT phosphorylation and inhibits tumorigenesis in mouse model.**

(**A**) The schematic diagram of orthotopic xenograft models in nude mice and relative treatments. (**B**) The statistics chart of liver weights in relative treatment groups. (**C**) The statistics chart of liver/body weight ratio in relative treatment groups. (**D**) The statistics chart of the number of number of liver nodules. (**E**) The gross images, HE staining and IHC staining of relative treatment groups. (**F**) The western blot results verified the expression levels of TMCO3 and other targets in nude mice.

**Supplementary Figure 8**

**
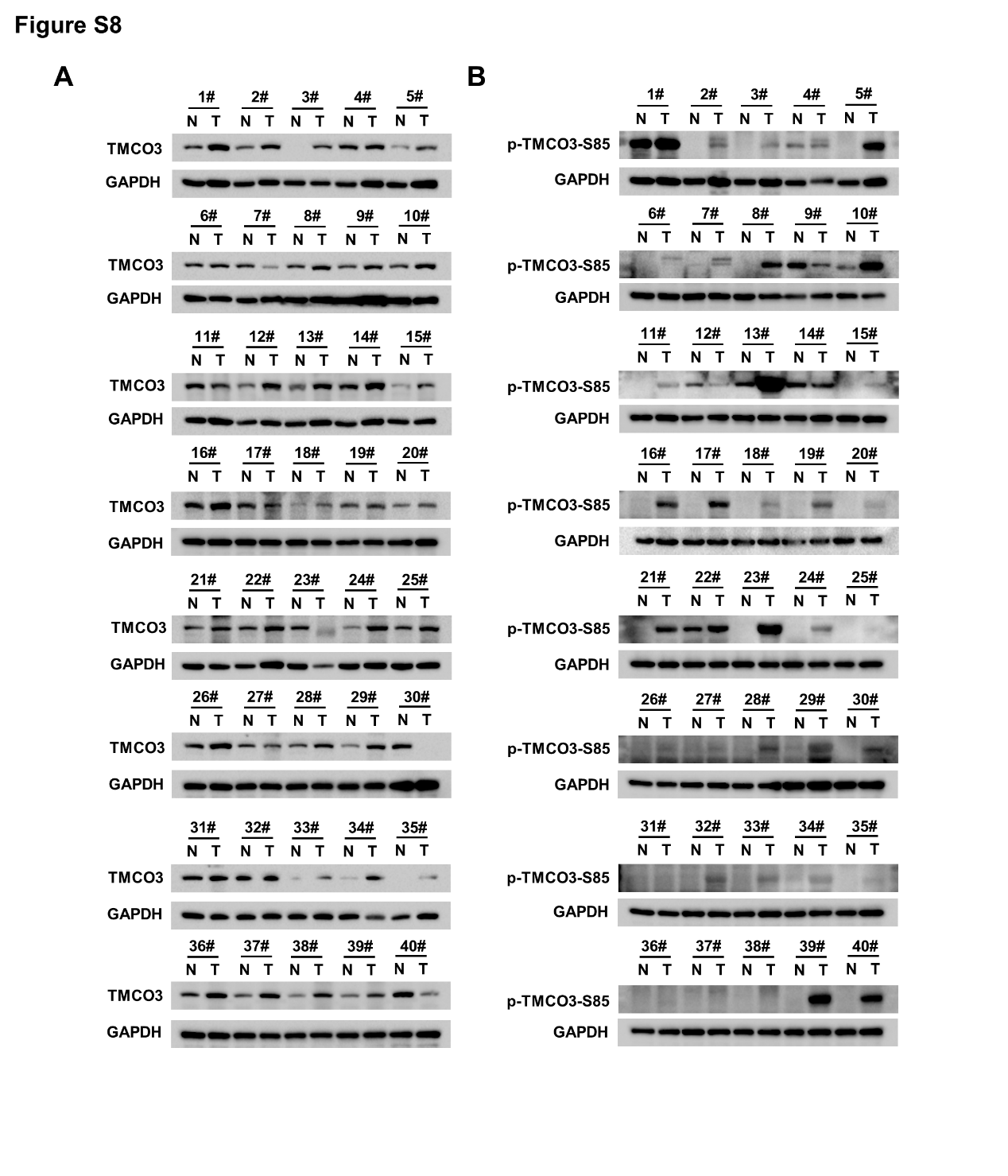
**

**Fig S8. The levels of TMCO3 and p-TMCO3-S85 are positively correlated with HCC and predict poor prognosis.**

(**A**) The western blot results showed the TMCO3 protein level in adjacent non-cancerous tissues or tumor tissues of 40 pairs of HCC patients. (**B**) The western blots results showed the P-TMCO3-S85 protein level in normal or tumor tissues of 40 pairs of HCC patients.

**Supplementary Tables**

|  |  |  |  |
| --- | --- | --- | --- |

**Supplementary Table 1. Correlation between TMCO3 and clinicopathological characteristics in HCC (n=123).**

| **Clinicopathological**  **variables** | **Relative TMCO3 Expression** | | **P value** |
| --- | --- | --- | --- |
|  | **Low 41** | **High 82** |  |
| **Gender**  Male  Female | 34  7 | 69  13 | 0.862 |
| **Age**  ≤50  > 50 | 24  17 | 36  46 | 0.125 |
| **AFP (ug/L)**  ≤20  > 20 | 9  32 | 24  58 | 0.387 |
| **GGT(u/l)**  ≤54  > 54 | 24  17 | 35  47 | 0.097 |
| **ALT(ng/ml)**  ≤75  >75 | 37  4 | 77  5 | 0.713 |
| **Cirrhosis**  No  Yes | 11  30 | 24  58 | 0.777 |
| **Tumor size (cm)**  ≤5  >5 | 17  24 | 28  54 | 0.427 |
| **Tumor encapsulation**  Complete  None | 18  23 | 52  30 | **0.039** |
| **Tumor number**  Single  Multiple | 28  13 | 69  13 | **0.042** |
| **Vascular invasion**  No  Yes | 29  12 | 67  15 | 0.166 |
| **Differentiation**  I- II  III-IV | 21  20 | 21  61 | **0.005** |
| **BCLC stage**  0+A  B+C | 25  16 | 53  29 | 0.691 |

**Supplementary Table 2. Multivariate analysis of factors associated with survival rates of 123 HCC patients in Tongji cohort.**

**Multivariate analysis (Survival)**

|  | **P value** | **HR** | **95% CI** |
| --- | --- | --- | --- |
|  |  |  |  |
| **TMCO3 expression** | **0.040** | 1.922 | **1.031-3.585** |
| **ALT** | **0.021** | 1.022 | **1.003-1.041** |
| **AST** | **0.024** | 0.984 | **0.970-0.998** |
| **AFP** | 0.657 | 0.883 | **0.509-1.531** |
| **Child-pugh score** | 0.430 | 2.364 | **0.279-20.005** |
| **Macrovascular invasion** | **0.016** | 3.003 | **1.224-7.367** |
| **Microvascular invasion** | 0.392 | 1.489 | **0.598-3.705** |
| **Tumor encapsulation** | 0.071 | 0.613 | **0.361-1.043** |
| **Tumor size** | 0.409 | 0.964 | **0.883-1.052** |
| **Cirrhosis** | 0.811 | 0.971 | **0.766-1.232** |
| **Tumor number** | **0.018** | 0.239 | **0.076-0.750** |
| **Differentiation** | 0.143 | 0.864 | **0.710-1.051** |

**Supplementary Table 3. List of all reagents used in this study.**

| **REAGENT or RESOURCE** | **SOURCE** | **IDENTIFIER** |
| --- | --- | --- |
| **Antibodies** | | |
| Anti-METTL3 Antibody | Proteintech | 15073-1-AP |
| Anti-METTL14 Antibody | Proteintech | 26158-1-AP |
| Anti-WTAP Antibody | Proteintech | 10200-1-AP |
| Anti-FTO Antibody | Proteintech | 27226-1-AP |
| Anti-GAPDH Antibody | Proteintech | 60004-1-Ig |
| Anti-IGF2BP2 Antibody | Proteintech | 11601-1-AP |
| Anti-TMCO3 Antibody | Abcam | ab154581 |
| Anti-Thiophosphate ester Antibody | Abcam | ab92570 |
| Anti-TMCO3 Antibody | Atlas | HPA048126 |
| Anti-AKT Antibody | CST | #4691 |
| Anti-Phospho-AKT (Thr308) Antibody | CST | #13038 |
| Anti-Phospho-AKT (Ser473) Antibody | CST | #4060 |
| Anti-Phospho-(Ser/Thr) Phe Antibody | CST | #9631 |
| Anti-c-MET Antibody | CST | #8198 |
| Anti-β-catenin Antibody | CST | #8480 |
| Anti-GST-Tag Antibody | CST | #2625 |
| Anti-Na+/K+-ATPase Antibody | ABclonal | A11683 |
| Anti-Phospho-TMCO3-S85 | ABclonal | E28609 |
| Anti-IGF2BP1 Antibody | ABclonal | A22246 |
| Anti-IGF2BP3 Antibody | ABclonal | A23295 |
| Anti-YTHDF1 Antibody | ABclonal | A23773 |
| Anti-YTHDC2 Antibody | ABclonal | A24219 |
| Anti-EIF3A Antibody | ABclonal | A0573 |
| Anti-mouse IgG for IP (HRP) | Vazyme | RA1009-01 |
| Anti-rabbit IgG for IP (HRP) | Vazyme | RA1008-01 |
| HRP, Goat Anti-Rabbit IgG | Abbkine | A21020 |
| HRP, Goat Anti-Mouse IgG | Abbkine | A21010 |
| Anti-FLAG Antibody | Sigma-Aldrich | F1804 |
| Anti-HA Antibody | Sigma-Aldrich | H6908 |
| **Other reagents** | | |
| FastPure Cell/Tissue Total RNA Isolation Kit V2 | Vazyme | RC112-01 |
| HiScript III RT SuperMix for qPCR | Vazyme | R323-01 |
| ChamQ Universal SYBR qPCR Master Mix | Vazyme | Q711-02 |
| BL21(DE3) competent cell | Vazyme | C504-02 |
| Fast-T1 competent cell | Vazyme | C505-02 |
| 2 × Phanta Flash Master Mix | Vazyme | P510-01 |
| Lipomaster 3000 Transfection Reagent | Vazyme | TL301-01 |
| CCK-8 Cell Counting Kit | Vazyme | A311-01 |
| Coomassie Blue Fast Staining Solution | Vazyme | E901-02 |
| Opti-MEM | Gibco | 31985070 |
| DMEM | HyClone | A003-4-1 |
| Fetal Bovine Serum | Gibco | 10091148 |
| MK2206 | Selleck | S1078 |
| Pictilisib | Selleck | S1065 |
| IGF-1 | PeproTech | 100-11 |
| Mem-PER™ Plus membrane protein extraction kit | ThermoFisher | 89842 |
| TSA Fluorescence Triple Staining Kit | ABclonal | RK05903 |
| Magna MeRIP m^6^A Kit | Sigma-Aldrich | 17-10499 |
| ATP-γ-S | Abcam | ab138911 |
| p-Nitrobenzyl mesylate | Abcam | ab138910 |
| 3X FLAG peptide | MedChamExpress | HY-P0319 |
| Anti-Flag Magnetic Beads | MedChamExpress | HY-K0207-1 |
| Actinomycin D | MedChamExpress | HY-17559 |
| Dimethylamiloride | MedChamExpress | HY-138866 |
| GST Purification Magbeads | Absin | Abs9902 |
| Protein A/G Magnetic Beads | Biolinkedin | L-1004 |
| PhosSTOP | Roche | 4906845001 |
| EDTA-free Protease Inhibitor | Roche | 4693132001 |
| RIPA lysis buffer | Meilunbio | MA0151 |
| IP lysis buffer | Meilunbio | MB9900 |
| PAGE Gel Quick Preparation Kit (10%) | YEASEN | 20325ES62 |
| Protein Marker | ThermoFisher | 26616 |
| PIP Strips | Echelon Biosciences | P-6001 |
| PI (3,4,5) P3 Beads | Echelon Biosciences | P-B345A |
| Kinase-Lumi^TM^ Chemiluminescent KinaseActivity Assay Kit | Beyotime | S0150S |

**Supplementary Table 4.** **Proteins bind with TMCO3 identified by IP-MS.**

| **Gene** | **Mw (kDa)** | **Length** | **Score** | **Unique sequence coverage (%)** | **Peptides** | **Unique peptides** |
| --- | --- | --- | --- | --- | --- | --- |
| ACTG1 | 41.793 | 375 | 323.31 | 4.5% | 20 | 1 |
| VIM | 53.652 | 466 | 323.31 | 65.5% | 42 | 37 |
| TUBA1A | 50.136 | 451 | 323.31 | 3.1% | 20 | 1 |
| TUBB4B | 49.831 | 445 | 323.31 | 2.7% | 22 | 1 |
| TMCO3 | 75.598 | 677 | 323.31 | 26.1% | 24 | 24 |
| ATP5F1B | 56.56 | 529 | 323.31 | 57.8% | 22 | 22 |
| AKT1 | 55.686 | 480 | 164.7 | 39.6% | 18 | 18 |
| PHGDH | 56.651 | 533 | 323.31 | 47.5% | 22 | 21 |
| ATP5F1A | 59.751 | 553 | 323.31 | 46.5% | 22 | 22 |
| HSP90AB1 | 83.264 | 724 | 323.31 | 28.9% | 39 | 21 |
| HSPA8 | 70.898 | 646 | 323.31 | 48.6% | 27 | 24 |
| RCN2 | 36.876 | 317 | 323.31 | 39.1% | 10 | 10 |
| ZNF326 | 65.654 | 582 | 323.31 | 41.9% | 19 | 19 |
| HSPD1 | 61.055 | 573 | 323.31 | 53.2% | 26 | 26 |
| C1QBP | 31.362 | 282 | 323.31 | 42.9% | 8 | 8 |
| HSPA1B | 70.052 | 641 | 323.31 | 25.6% | 24 | 14 |
| RPN2 | 69.284 | 631 | 323.31 | 44.8% | 19 | 19 |
| DBN1 | 71.429 | 649 | 323.31 | 41.8% | 18 | 18 |
| ATP2A2 | 114.757 | 1042 | 323.31 | 33.7% | 32 | 32 |
| HNRNPH1 | 49.229 | 449 | 323.31 | 31.8% | 16 | 10 |
| SPTAN1 | 284.539 | 2472 | 323.31 | 57.7% | 123 | 122 |
| SPTBN1 | 274.609 | 2364 | 323.31 | 52.5% | 102 | 96 |
| ACTN4 | 104.854 | 911 | 323.31 | 37.2% | 42 | 26 |
| HNRNPUL2 | 85.105 | 747 | 323.31 | 35.5% | 25 | 25 |
| PRKDC | 469.089 | 4128 | 323.31 | 33.4% | 116 | 115 |
| MYH10 | 228.999 | 1976 | 323.31 | 31.6% | 69 | 55 |
| DSP | 331.774 | 2871 | 323.31 | 34.7% | 83 | 80 |
| PLEC | 531.791 | 4684 | 323.31 | 36.1% | 145 | 141 |
| MYH9 | 226.532 | 1960 | 323.31 | 31.2% | 61 | 49 |
| CAD | 242.984 | 2225 | 323.31 | 33.6% | 50 | 49 |
| NEFM | 102.472 | 916 | 323.31 | 29.9% | 22 | 20 |
| GCN1 | 292.71 | 2671 | 323.31 | 26.2% | 63 | 63 |
| DYNC1H1 | 532.408 | 4646 | 323.31 | 29% | 107 | 105 |
| DDOST | 50.801 | 456 | 315.72 | 37.7% | 14 | 14 |
| XPO1 | 123.386 | 1071 | 307.6 | 38.7% | 35 | 35 |
| VDAC1 | 30.773 | 283 | 285.93 | 58.7% | 14 | 13 |
| FLNA | 280.739 | 2647 | 282.35 | 31.3% | 53 | 52 |
| MYO6 | 149.691 | 1294 | 280.83 | 38.9% | 36 | 36 |
| SLC3A2 | 67.994 | 630 | 279.36 | 36.3% | 21 | 20 |
| DDX3X | 73.243 | 662 | 277.48 | 35.6% | 19 | 18 |
| PSMC5 | 45.626 | 406 | 273.38 | 53% | 22 | 22 |
| IARS1 | 144.498 | 1262 | 271.84 | 35.4% | 35 | 35 |
| VDAC2 | 31.567 | 294 | 269.35 | 55.8% | 12 | 12 |
| MTHFD1 | 101.531 | 935 | 268.97 | 38.7% | 29 | 29 |
| DHX9 | 140.958 | 1270 | 262.49 | 28.3% | 29 | 28 |
| PCNA | 28.769 | 261 | 262.23 | 62.8% | 11 | 11 |
| HNRNPM | 77.516 | 730 | 251.38 | 37.3% | 23 | 22 |
| FLNB | 278.164 | 2602 | 244.18 | 30.1% | 51 | 48 |
| DARS1 | 57.136 | 501 | 244.08 | 50.3% | 21 | 21 |
| PPP1R9B | 89.334 | 817 | 243.17 | 32.4% | 20 | 19 |
| CAPZA1 | 32.923 | 286 | 237.86 | 51.4% | 10 | 8 |
| EPRS1 | 170.591 | 1512 | 237 | 33.6% | 39 | 38 |
| HSPA9 | 73.681 | 679 | 233.68 | 40.5% | 19 | 19 |
| GOLGA2 | 113.086 | 1002 | 229.98 | 28.4% | 18 | 17 |
| CCT2 | 57.488 | 535 | 226.46 | 47.3% | 19 | 19 |
| AMOT | 118.085 | 1084 | 224.88 | 31.5% | 26 | 24 |
| MCM7 | 81.308 | 719 | 222.69 | 34.8% | 22 | 22 |
| IPO5 | 123.63 | 1097 | 222.18 | 23.8% | 19 | 18 |
| TCP1 | 60.344 | 556 | 221.53 | 43.2% | 20 | 19 |
| HNRNPK | 50.976 | 463 | 221.5 | 35.9% | 13 | 12 |
| MCM4 | 96.558 | 863 | 219.79 | 39.3% | 24 | 24 |
| PSMA5 | 26.411 | 241 | 218.44 | 56.4% | 9 | 9 |
| RCN1 | 38.89 | 331 | 218.35 | 47.7% | 14 | 14 |
| MARS1 | 101.116 | 900 | 217.95 | 35% | 23 | 23 |
| HNRNPF | 45.672 | 415 | 212.51 | 32.5% | 10 | 9 |
| MCM3 | 90.981 | 808 | 212.47 | 37.1% | 24 | 23 |
| PSMC4 | 47.366 | 418 | 209.77 | 51.7% | 21 | 21 |
| GAN | 67.638 | 597 | 209.7 | 44.6% | 21 | 21 |
| KPNB1 | 97.17 | 876 | 207.1 | 30.6% | 19 | 19 |
| NUP205 | 227.922 | 2012 | 207.05 | 16.4% | 25 | 25 |
| ATP1A1 | 112.896 | 1023 | 205.79 | 29% | 24 | 24 |
| RUVBL2 | 51.157 | 463 | 203.11 | 51% | 21 | 21 |
| AIFM1 | 66.901 | 613 | 202.82 | 32% | 15 | 15 |
| DDB1 | 126.968 | 1140 | 201.95 | 36.5% | 38 | 36 |
| EEF1A1 | 50.141 | 462 | 201.42 | 16% | 16 | 8 |
| TRIM21 | 54.17 | 475 | 195.97 | 43.2% | 18 | 18 |
| CCT4 | 57.924 | 539 | 195.32 | 35.3% | 16 | 14 |
| TUBB | 49.671 | 444 | 194.77 | 16% | 21 | 4 |
| LMNB1 | 66.408 | 586 | 194.23 | 37% | 17 | 17 |
| XPOT | 109.964 | 962 | 193.36 | 32.6% | 25 | 25 |
| LRPPRC | 157.905 | 1394 | 192.53 | 32.6% | 40 | 39 |
| CAND1 | 136.376 | 1230 | 191.72 | 23.3% | 26 | 23 |
| CCT5 | 59.671 | 541 | 191.21 | 46.4% | 24 | 24 |
| UQCRC2 | 48.443 | 453 | 191.14 | 40.2% | 12 | 12 |
| NPM1 | 32.575 | 294 | 190.99 | 43.9% | 9 | 9 |
| RUVBL1 | 50.228 | 456 | 188.46 | 63.2% | 21 | 21 |
| MDN1 | 632.82 | 5596 | 185.57 | 13.5% | 53 | 53 |
| COPA | 138.346 | 1224 | 185.45 | 34.2% | 33 | 33 |
| FASN | 273.427 | 2511 | 185.36 | 27% | 46 | 46 |
| PSMC3 | 49.204 | 439 | 183.71 | 47.8% | 18 | 17 |
| CLTC | 191.615 | 1675 | 183.46 | 30.4% | 38 | 38 |
| IMMT | 83.678 | 758 | 182.62 | 42.2% | 21 | 21 |
| MSH6 | 152.786 | 1360 | 181.72 | 23.7% | 24 | 24 |
| PSMD1 | 105.836 | 953 | 181.61 | 33.3% | 24 | 22 |
| PRPF8 | 273.6 | 2335 | 181.61 | 26.7% | 48 | 48 |
| TMPO | 75.492 | 694 | 179.48 | 26.8% | 18 | 12 |
| PSMD2 | 100.2 | 908 | 178.28 | 37.1% | 27 | 25 |
| NCLN | 62.974 | 563 | 177.41 | 34.6% | 16 | 15 |
| NPEPPS | 103.276 | 919 | 175.25 | 33% | 23 | 23 |
| IRS4 | 133.768 | 1257 | 174.94 | 21.1% | 17 | 17 |
| TOMM22 | 15.522 | 142 | 172.77 | 76.8% | 6 | 6 |
| HNRNPU | 90.584 | 825 | 172.01 | 27.9% | 19 | 18 |
| DDX17 | 80.272 | 729 | 171.77 | 26.7% | 23 | 17 |
| RTCB | 55.21 | 505 | 171.72 | 35.8% | 15 | 14 |
| HSPA5 | 72.333 | 654 | 171.31 | 37.5% | 19 | 18 |
| RPN1 | 68.569 | 607 | 169.79 | 46.5% | 20 | 20 |
| KRT18 | 48.058 | 430 | 166.48 | 44.7% | 16 | 15 |
| YWHAE | 29.174 | 255 | 160.49 | 49.4% | 12 | 10 |
| PSMC2 | 48.634 | 433 | 158.03 | 51.5% | 22 | 22 |
| NUP93 | 93.488 | 819 | 156.56 | 43.8% | 28 | 28 |
| DNAJA1 | 44.868 | 397 | 155.24 | 41.6% | 13 | 13 |
| EEF2 | 95.338 | 858 | 154.12 | 31% | 23 | 22 |
| COPG1 | 97.718 | 874 | 153.53 | 23.5% | 17 | 15 |
| ABCD3 | 75.476 | 659 | 152.98 | 24.7% | 16 | 15 |
| COPE | 34.482 | 308 | 151.74 | 41.6% | 9 | 9 |
| TRMT10C | 47.347 | 403 | 151.73 | 46.7% | 15 | 15 |
| SNCA | 14.46 | 140 | 150.96 | 42.9% | 5 | 5 |
| QARS1 | 87.799 | 775 | 150.74 | 33.2% | 19 | 19 |
| YWHAQ | 27.764 | 245 | 149.06 | 40.4% | 10 | 8 |
| EEF1G | 50.119 | 437 | 148.25 | 30.4% | 14 | 14 |
| MCM6 | 92.889 | 821 | 147.44 | 31.3% | 23 | 22 |
| YME1L1 | 86.455 | 773 | 146.12 | 26.1% | 14 | 14 |
| ACTN1 | 103.058 | 892 | 145.72 | 30.7% | 34 | 19 |
| EMD | 28.994 | 254 | 144.47 | 46.1% | 9 | 9 |
| NUP210 | 205.111 | 1887 | 144.23 | 16.2% | 22 | 22 |
| SLC25A11 | 34.062 | 314 | 143.4 | 39.2% | 10 | 10 |
| COPB1 | 107.142 | 953 | 143.34 | 38.1% | 30 | 29 |
| SNRNP200 | 244.508 | 2136 | 143.11 | 21.8% | 37 | 36 |
| MSH2 | 104.743 | 934 | 142.66 | 26.9% | 20 | 19 |
| TUBA1C | 49.895 | 449 | 140.71 | 15.1% | 19 | 4 |
| TUBB8 | 49.776 | 444 | 140.65 | 7.4% | 10 | 2 |
| CDK1 | 34.095 | 297 | 140 | 52.2% | 15 | 13 |
| ATXN10 | 53.489 | 475 | 138.54 | 32.6% | 12 | 12 |
| CCDC47 | 55.874 | 483 | 138.24 | 27.7% | 10 | 10 |
| DNAJA2 | 45.746 | 412 | 138 | 25% | 9 | 9 |
| RBBP7 | 47.82 | 425 | 136.66 | 24.5% | 11 | 7 |
| PSMD3 | 60.978 | 534 | 136.24 | 36% | 18 | 18 |
| ALDH1B1 | 57.249 | 517 | 135.62 | 25.1% | 12 | 12 |
| SEC13 | 35.541 | 322 | 135.57 | 40.4% | 8 | 8 |
| IPO9 | 115.963 | 1041 | 134.04 | 19.9% | 15 | 15 |
| AIF1L | 17.068 | 150 | 132.61 | 55.3% | 8 | 7 |
| DDX5 | 69.148 | 614 | 132.05 | 23.1% | 19 | 14 |
| PCBP1 | 37.498 | 356 | 131.25 | 28.7% | 9 | 6 |
| TJP1 | 195.459 | 1748 | 131.06 | 18.2% | 23 | 22 |
| TMOD3 | 39.595 | 352 | 130.95 | 40.9% | 13 | 12 |
| RPLP2 | 11.665 | 115 | 129.85 | 70.4% | 8 | 7 |
| GLB1 | 76.075 | 677 | 129.62 | 28.1% | 15 | 15 |
| TUFM | 49.875 | 455 | 129.55 | 41.1% | 17 | 15 |
| CCT8 | 59.621 | 548 | 129.42 | 43.1% | 20 | 20 |
| IGF2BP1 | 63.481 | 577 | 128.03 | 24.8% | 15 | 12 |
| HACD3 | 43.16 | 362 | 127.92 | 32.6% | 10 | 10 |
| IPO7 | 119.517 | 1038 | 126.34 | 24.1% | 17 | 17 |
| HNRNPA0 | 30.841 | 305 | 125.44 | 38.4% | 6 | 6 |
| PGRMC1 | 21.671 | 195 | 125.13 | 40.5% | 7 | 6 |
| CIAO2B | 17.663 | 163 | 124.91 | 67.5% | 6 | 6 |
| DIS3 | 109.003 | 958 | 124.47 | 30.1% | 23 | 22 |
| HSD17B12 | 34.324 | 312 | 124.42 | 43.3% | 12 | 12 |
| DDX1 | 82.432 | 740 | 123.65 | 31.2% | 17 | 17 |
| RPL37A | 10.275 | 92 | 123.43 | 19.6% | 1 | 1 |
| SKIC3 | 175.486 | 1564 | 123.25 | 14.6% | 17 | 17 |
| HNRNPDL | 46.438 | 420 | 123.23 | 17.1% | 10 | 9 |
| NDUFS1 | 79.468 | 727 | 122.99 | 39.3% | 18 | 18 |
| FAM98A | 55.273 | 518 | 122.58 | 18.7% | 8 | 6 |
| SEC22B | 24.741 | 215 | 121.99 | 49.3% | 8 | 8 |
| CFL1 | 18.502 | 166 | 121.74 | 56% | 7 | 7 |
| STT3A | 80.53 | 705 | 120.94 | 16.2% | 11 | 9 |
| RPLP0 | 34.274 | 317 | 120.21 | 52.1% | 11 | 11 |
| AGPS | 72.912 | 658 | 119.97 | 37.5% | 17 | 16 |
| SF3B3 | 135.577 | 1217 | 119.87 | 26% | 23 | 23 |
| AHSA1 | 38.274 | 338 | 119.82 | 53.3% | 14 | 13 |
| CSDE1 | 88.885 | 798 | 119.36 | 25.4% | 16 | 16 |
| MYL12A | 19.794 | 171 | 119 | 62.6% | 8 | 8 |
| SFXN1 | 35.619 | 322 | 117.83 | 21.1% | 5 | 5 |
| SMC4 | 147.182 | 1288 | 117.4 | 23.1% | 24 | 23 |
| HSP90B1 | 92.469 | 803 | 116.75 | 24.2% | 19 | 17 |
| LARS1 | 134.466 | 1176 | 116.72 | 29.6% | 28 | 26 |
| PSMC6 | 44.173 | 389 | 116.62 | 39.8% | 13 | 12 |
| TUBGCP2 | 102.534 | 902 | 116.57 | 29.4% | 19 | 18 |
| CALU | 37.107 | 315 | 115.72 | 57.1% | 14 | 14 |
| SSR3 | 21.081 | 185 | 115.52 | 7.6% | 1 | 1 |
| GAPDH | 36.053 | 335 | 115.51 | 54.6% | 11 | 11 |
| HNRNPA1 | 38.747 | 372 | 114.8 | 25% | 8 | 8 |
| MYL6 | 16.93 | 151 | 113.19 | 29.1% | 8 | 5 |
| CCT6A | 58.024 | 531 | 112.61 | 20.2% | 9 | 9 |
| PPP1CA | 37.512 | 330 | 112.28 | 9.7% | 14 | 3 |
| RPL10 | 24.577 | 214 | 111.74 | 36% | 8 | 8 |
| PSMD4 | 40.737 | 377 | 111.57 | 32.6% | 10 | 10 |
| SMC3 | 141.542 | 1217 | 111.08 | 24.1% | 23 | 23 |
| PABPC1 | 70.671 | 636 | 110.08 | 16.8% | 16 | 11 |
| SMC2 | 135.656 | 1197 | 109.94 | 22.8% | 24 | 24 |
| SPTLC1 | 52.744 | 473 | 109.84 | 21.4% | 6 | 6 |
| MYBBP1A | 148.855 | 1328 | 109.59 | 20.5% | 22 | 22 |
| EIF4A1 | 46.154 | 406 | 109.51 | 38.2% | 15 | 14 |
| CSE1L | 110.417 | 971 | 108.93 | 23.2% | 20 | 20 |
| GART | 107.767 | 1010 | 108.87 | 30% | 21 | 21 |
| PRDX1 | 22.11 | 199 | 108.14 | 55.3% | 11 | 9 |
| XPO5 | 136.311 | 1204 | 108.02 | 15.4% | 13 | 13 |
| SERPINH1 | 46.441 | 418 | 106.7 | 43.8% | 16 | 16 |
| NSF | 82.594 | 744 | 106.01 | 30.1% | 21 | 20 |
